# Supplementary material for: Regional and institutional trends in assessment for academic promotion
Source: Nature. 2025 Jan 22;638(8050):459–68. doi: 10.1038/s41586-024-08422-9 (PMC11821531; doi:10.1038/s41586-024-08422-9)
Supplement: Supplementary file 1 — Supplementary Methods, Tables 1–11 and References. [file 41586_2024_8422_MOESM1_ESM.pdf]

nature portfolio

https://doi.org/10.1038/s41586-024-08422-9

# Supplementary information

# Regional and institutional trends in assessment for academic promotion

In the format provided by the authors and unedited

# 1. SUPPLEMENTARYMETHODS

## 1.1. Study design and definition of methodology

This study was initiated by some of the authors who, in 2016, sought to understand what matters for institutions globally in terms of scientific excellence and to which extent our global research culture is influenced by metrics. The team hypothesised that these factors could be objectively studied by looking at the assessment criteria used in institutional promotion policies. Leveraging the global platform provided by the Global Young Academy (GYA), the team launched a call to all members and alumni (representing 100 countries) to request promotion policies from their respective institutions or countries.

As a result of the call, the team collected 42 promotion documents from various world regions and began defining a methodological framework to capture and quantify data embedded on the documents, enabling global comparison. This process required clear definitions of which data to include, as well as how these data should be coded to ensure reproducibility and reusability. These details are expanded in our protocol, in a point-by-point manner, shared with our replication package (data availability section) and throughout Section 1 of the Supplementary material. However, here we briefly narrate the rationale and context.

With this empirical exercise, it became evident that academic careers are not uniform worldwide and that the working conditions of researchers are significantly different. To address these differences, we imposed certain limitations on the way we sourced, interpreted, and recorded data. First, we limited our data sourcing to promotion policies for full professors, as this role was the only one universally present in virtually all documents describing policies (see Section 1.2). We retained only documents that offer academic paths or include posts covering both research and teaching, while acknowledging the fact that some institutions offer different career tracks that may not be comparable. Additionally, after conducting a round of interviews with representatives from various institutions to explore data sourcing options, the team concluded that a strict and consistent interpretation of the data was essential, limiting our study to accounting for the presence or absence of specific assessment criteria. The definition of our data units and the adopted procedures for their inclusion and categorisation are detailed in Section 1.2.3.

To define the promotion criteria to be studied, the team listed and scored the criteria based on their frequency of occurrence, from most commonly used to rarely observed. A subset of criteria consistently appeared across policies and was thus defined as the core criteria for the study (see Section 1.4.1). Clear definitions were then established for the inclusion of each criterion, with the team adhering strictly to the definitions presented in Table S1. Additionally, we categorised the criteria using the procedures outlined in sections 1.4.2 and 1.5, Table S2 to the categories shown in Extended Data Table 3. This process led to the development of a

standardised data coding template, which we used to encode whether a criterion was present or absent (see Section 1.6 and the replication package).

At a later stage, to improve global coverage and incorporate reviewer advice (including policy updates), we recruited team members to participate in data sourcing, coding, and interpretation of the criteria. We established geographical teams and developed a standardised matrix template as well as data coding guidelines. Several meetings were held, and recordings exemplifying the interpretation and coding of data were distributed (see metadata in our replication package for examples; videos cannot be shared due to data privacy). Each author was given a package with the necessary information and dedicated space to store and code the data. Team leaders were responsible for distributing tasks among members and consolidating the data into regional sub-datasets. To ensure accurate coding, each policy was reviewed by two co-authors. Team leaders mediated any differences in interpretation and, if necessary, engaged with project leads to resolve discrepancies or amend the definitions of criteria. To facilitate this process, an evidence cell was included next to each encoded criterion, allowing authors to document the location of the reference and quote the exact text extracted (See section 1.6). The data shared with this manuscript includes all this information, including the URL of the policy, the author who coded the policy, the authors who reviewed it, and the evidence supporting the coding of each criterion as present or absent (see replication package).

The data analysis initially began with descriptive statistics, but as certain patterns emerged, a principal factor analysis was proposed to identify underlying trends. Later, following the advice of peer reviewers, we incorporated a regression analysis to uncover any underlying confounding elements.

## 1.2. Sourcing the documents describing promotion policies

### *1.2.1. Sampling strategy*

For obtaining a global sample of policies for promotion to (full) professor, one promptly encounters conflicting goals. We adopted a purpose-oriented pragmatic approach that makes our study well-suited for spotting key trends, but we do not aim at being fully comprehensive. What primarily matters for our inferred conclusions is to capture global diversity and to find systematic patterns. It is worth keeping in mind that specific researchers do not face the average, but some diversity, and (within limitations) they are able to make conscious decisions on institutions they would want to target for seeking employment, while their situation is certainly different depending on where in the world they are located.

Unlike studies that focus on single countries or regions (typically advanced economies) [e.g., 1], we cannot use an official list of academic or research institutions from which to randomly draw a certain number of sampled units. Such a list is unavailable at the global level and non-existent in several countries or regions.

Therefore, stratified random sampling is impossible for a work like ours, that tries to convey a global picture. We thus leveraged on members and alumni of the GYA to source documents not only from their own institutions, but in a way of snowball sampling also from other institutions they know or have a relation with, or by involving their academic network. The advantages brought upon by this network was having native speakers of different languages and the knowledge of regional institutional or governmental frameworks facilitating the sourcing of documents from different institutions worldwide.

While the only possibility in practice, we do not regard this snowball sampling as engendering any specific bias. Specifically, neither the composition of the group of people sourcing the policy documents nor any filtering of documents was informed by the promotion criteria that are the object of our study. We included each and every sourced document that was sufficiently clear and comprehensive for us to identify the absence or presence of specific criteria (see sect. 1.3). While our set of policy documents does not constitute a random sample, it can reasonably be considered as an unbiased one for the purposes of this study.

In fact, our sampling specifically aimed at maximising the reach and scope, so that our sample is as wide and diversified as possible, in terms of countries covered, areas of the world, and economic status. Consequently, as highlighted in the main text, we obtained the largest dataset (to our knowledge) of promotion criteria globally to date. In this manner, we arrived at documents from 190 academic institutions and 58 government agencies in 121 countries (one agency being supra-national).

### *1.2.2. Sampling periods*

The first sampling wave took place between 2016 and 2018, during which 46 policies were collected and then used to inform our methodological framework. A second wave of sampling occurred from 2018 to 2021, yielding 159 policies from 56 countries, which were analysed to produce the first version of this study, available as a pre-print [2]. After the reviewer advice and noticing that many policies have an updating cycle of 5 or 10 years, we drove a third round of sourcing in 2023 for the present analysis, to check for updates in the policies collected and new policies to expand our global reach. From this exercise, we sourced 440 policies, of which 300 (56%) were implemented after 2020, 148 (28%) after 2015, 62 (12%), before 2015 and for 22 (4%) policies this information was not disclosed. Furthermore, based on the evidence gathered, 460 (87%) of the policies in our sample were applicable as of Dec 2023. While we could not gather any evidence of updates or revisions from the remaining policies, most of these policies were known to apply in 2020 and are very likely to still apply in 2024, as no replacement was found. Therefore, we are confident that our sample closely reflects the present state (2024).

### *1.2.3. Sample composition and access*

We consider each distinct set of assessment criteria applied for a promotion process to constitute a "policy" for promotion. Some institutions or agencies distinguish different career tracks or academic disciplines, and therefore there can be several policies within the same institution or agency. In total, our dataset covers 532 policies of which 426 policies (78%) were sourced from public websites, 106 policies (20%) were sourced through the co-authors' networks and 12 policies (2%) were obtained via an official request made through the GYA office. We share the respective URLs of policies found online in our dataset. All publicly available documents (78%) were accessed through corresponding websites, and the others were obtained and managed as hard copies in PDF files.

### *1.2.4. Language*

The documents collected were written in 27 languages, namely Albanian, Amharic, Arabic, Armenian, Bosnian, Cambodian, Chinese (simplified/traditional), Croatian, Dutch, English, French, Greek, Hebrew, Hindi, Indonesian, Italian, Malay, Nepali, Polish, Portuguese, Russian, Spanish, Thai, Turkish, Ukrainian, Uzbek, and Vietnamese. To allow a consistent interpretation, we used a mix of two approaches:

i. Translation of the document to English, using both translation software (e.g. Google Translate) and the help of GYA members who are fluent speakers of the language of interest. Given that machine translations are not always accurate, we explicitly cross-verified the accuracy of the translated documents. If documents came in non-machine-readable formats, we also applied Optical Character Recognition (OCR) tools for machine translations.

ii. Ensuring that the document is reviewed for criteria by at least two team members familiar with the language.

### *1.2.5. General quality control (Eligibility Criteria)*

We made sure not to have duplicates of the same policy received from different sources. Specifically, whenever different academic institutions reported identical policies, we took that as indication that the policy was set at national rather than institutional level (as described in sect. 1.2.1). Moreover, we dismissed any policies that are vague on the applied criteria (defined in sect. 1.3.1) and do not provide clear definitions. This particularly includes policies that never go beyond mentioning buzzwords like "excellence in research", "leadership", or "international visibility" and thereby leave it to reviewers or panels how to arrive at a judgement, with this process remaining inaccessible to us.

Moreover, we found that the Art and Creative Works disciplines stand out from other academic fields by focusing on quite different outputs or achievements. We decided not to add further detail by properly

categorising these, but for reasons of simplicity and coherence, we restricted our attention to the sciences and humanities, classified as described in section. 1.2.4.

## 1.3. Defining sub-samples

Our dataset was not obtained through a stratified sampling procedure, as we collected all documents that spell out the criteria for promotion to “full professor”. For the purpose of differential analysis, we categorised the policies by their country and related region, their scope (“institutional” or “national” policies), career tracks, and academic disciplines. Specifically, to account for possible systematic differences, we split our sample with regard to the Global North and Global South, as well as with regard to economic status according to the official nomenclature of international development institutions.

### *1.3.1. Policy scope*

Universities have different degrees of autonomy, even within the same country. Consequently, policies for academic promotion or parts of these policies are sometimes defined centrally by government agencies rather than by an institution itself. We therefore distinguish policies by whether these were developed by and apply to a specific academic or research institution (“institutional policies”) or whether these come from government agencies and apply equally to several institutions across the country (“national policies”).

National policies were found to fall within the remit of ministries that cover higher education, science, technology, innovation, research, or human resources development, and some were issued through related government agencies, namely:

i. *Government agencies under a universities, education, or higher education portfolio:* National Agency for Quality Assessment and Accreditation of Spain (ANECA), National Universities Commission (Nigeria), Commission of University Education (Kenya), Supreme Council of Universities (SCU, Egypt), Higher Education Council (CES, Ecuador), National Council for Science, Higher Education and Technological Development (NVZVOTR, Croatia), Higher Education Commission (HEC, Pakistan), Higher Education Authority (HEA, Zambia) etc.

ii. *Government agencies under a research, innovation, science, and/or technology portfolio:* National Research Foundation (NRF, South Africa), Department of Scientific Research and Technological Development (SENACYT, Panama), National Scientific and Technical Research Council (CONICET, Argentina), National Council of Science and Technology (CONACYT, Paraguay), National Council of Science, Technology and Technological Innovation (CONCYTEC, Peru) etc.

iii. *Other government agencies*: University Grants Commission (Sri Lanka), Department of Budget and Management (DBM, The Philippines), National Research and Innovation Agency (Indonesia), etc.

Some institutional policies were found to explicitly refer to national policies. In several cases, a national policy outlines basic requirements for promotion, while institutions can add additional criteria. Sometimes, national policies impose a specific scoring or ranking system, e.g. the National Research Foundation (South Africa) or the Department of Budget and Management (The Philippines). Whenever an institutional policy adopts parts of a national policy, we mark the respective criteria as mentioned by the institutional policy.

We considered any supra-national policy as a national policy for every country it applies to, namely the African and Malagasy Council for Higher Education (CAMES), which coordinates the higher education and research systems in francophone Africa for 22 countries, defining policies for 5 disciplines (see Extended data Table 1).

### *1.3.2. Country and region*

To establish the geographical distribution of the surveyed policies (as shown in Figure 1, main article), we followed the United Nations Geoscheme classification [3], which groups countries and territories into major continental regions, such as Africa, Asia, Europe, Latin America and the Caribbean, Northern America, and Oceania. With regard to transcontinental countries, we grouped the Russian Federation and Türkiye with Europe; Kazakhstan with Asia; and Egypt with Africa. Armenia was also grouped with Europe. The figure also provides full details on the number of policies by country and region. We adopted the World Bank data classification as reference for categorising the per-capita income of countries [4] and the UNSD 2018 classification to distinguish Global North and Global South [5]. See the number of policies per region in Extended Data Table 1.

### *1.3.3. Career tracks*

Promotion policies frequently consider different career tracks, distinguished by how much effort and weight is given to commitments in research, teaching, or other professional activities, including institutional leadership or clinical roles. Some policies refer to specific weights for assessment, while others do not, and instead just require minimal standards in each category to be met. Usually, there is a standard academic track, covering both research and teaching responsibilities, with weights or expectations however differing across institutions or organisations. We identified this standard academic track for each of the policies and adopted its set of criteria as the default for our analysis, thereby allowing and capturing some diversity on what is understood as the role of "professor" on standard academic track. Besides the standard academic track (research & teaching), a substantial number of policies (48%) consider a research-focused track, a teaching-focused track, or a clinical track. Some countries, specifically the United States, distinguish between tenure track and non-tenure track, but rather than following that classification, which is not a global feature, we matched the respective policies to our four track profiles. We also distinguish by an indicator whether a policy

is specific to the role of (full) professor (1) or whether it replies to researchers more generally or does not explicitly specify (0). See the corresponding number of policies per track in Extended Data Table 1.

#### *1.3.4. Disciplines*

Besides track-specific evaluations, we also came across discipline-specific criteria in our sample of promotion policies. Therefore, we consider four broad fields of research: *(1)* 'Natural Sciences', *(2)* 'Engineering and Technology', *(3)* 'Medical and Health Sciences', and *(4)* 'Social Sciences and Humanities', following the revised OECD classification 2006/7 [6], as well as *(5)* 'General' for policies that do not refer to specific disciplines or did not show any differences across disciplines. We did not consider 'Agricultural Sciences' due to lack of data (the respective policies have been merged into the natural sciences) and we combined 'Social Sciences' and 'Humanities' into a single category. We did not consider scholarship in the Arts and Creative works given substantial differences in the nature and relevant outputs of this field in comparison to other disciplines. For each policy specific to one or more disciplines, we noted the respective group(s) they belong to, with this policy potentially straddling two or more groups. See number of policies included in Extended Data Table 1.

### 1.4. Choice of evaluation criteria

#### *1.4.1. Definition of criteria*

The definition of categories is always somewhat subjective, and our categorisation considered the need to be a reasonable reflection of key features of the policies as well as their diversity, to be comparable in different national and regional contexts (i.e. not relating to divergent national of regional policy frameworks or having different meanings in various environments), and not to spread out into too much detail. We also required criteria to be sufficiently distinct, i.e. not overlapping too much with other criteria. We also did not adopt criteria that correspond to mandatory formal qualifications for the role (e.g. holding a doctoral degree) or broad and unspecific traits (e.g. a good moral character), found in some policies.

In our pilot study, we identified 30 distinct typical evaluation criteria that occurred consistently across a substantial number of policies — listed and described in detail in Table **S1**. We specifically considered teaching as a single criterion rather than delving into the complex details of teaching assessment, because it was found to be highly variable across policies and difficult to categorise with our current methods and scope. Furthermore, while there may be various outputs listed in different policies, we focused our study on criteria relating to those that were consistently mentioned across the policies. These primarily refer to "publications" or "intellectual property".

**Table S1. Evaluation categories for promotion to professorship.** List of the criteria extracted from the promotion policies, included in this study and their description or specific indications noted from the documents.

| Criteria | Description provided on surveyed policies |
|---|---|
| Number of publications | Specific mentioning of the *quantity* of research publications. If the policy only mentions "publications'', then it is not considered under this category because the quantity may not be the focus of evaluators. Publications could in principle be any textual outputs available to anybody (potentially for payment), including in particular: journal articles, conference papers, books, book chapters, monographs, policy briefs, pre-prints, newspaper, or magazine articles. These usually do *not* include any material that is proprietary or access-restricted to specific groups, such as funding applications or consulting reports. However, policies might use the term "publication" in a narrower sense. |
| Recent publications | The *quantity* of research publications within a specified timeframe, often referred to as "number of publications in current position", "number of publications after the last promotion", or "number of publications in the last x years", where some number x is given. |
| Patents | Any form of registered intellectual property (incl. patents, utility models or registered designs) regardless of how these are evaluated, e.g. by number or arising income. They might be granted or filed. Some policies also include patents as type of publication. |
| Citations | All measures that are based on citations of research outputs, most frequently the cumulative number of citations or the h-index, calculated on the basis of data sources like Web of Science, Scopus, or Google Scholar. |
| Journal indexing | Restriction of what is counted as qualifying "publication" to a specific list of journals or applying credit points according to such a list. This includes referring to indexed journals as well as to any journal ranking, be it based on the Journal Impact Factor (JIF), quartiles (or other percentiles) of Journal Citation Reports (JCR), Science Citation Index, Ei Compendex, Scopus, SCImago Journal Rank (SJR), SciELO, or similar databases, or on any other compiled list of journals that are perceived to have high standing or meet other defined criteria. |
| Number of authors | Explicit consideration of the number of authors of a given research publication. This might include applying weights (including zero) in relation to the number of authors. |
| Authorship order | Explicitly refers to the position of the applicant on the author list or a specific author role (e.g. corresponding or senior author), for which weighting schemes might be applied. |
| Non-metric journal quality | Evidence of publications in journals that are perceived as well-recognised by the evaluator (without referring to any journal impact metric) or that fulfil objective criteria that can be seen as indicators of quality, such as having a trustworthy expert peer-review college. |
| Role of authors | Assessing the specific contributions of the applicant to the published research works as well as their significance, rather than trying to make a guess from the position or a nominal role (e.g. corresponding or senior author) on the author list. This might include asking candidates for a narrative description or looking at the contribution sections of publications (if such exist). |
| Non-metric quality of publications | An assessment of quality based on the personal judgement of the evaluator based on reading (a selection of) the research outputs. This might be further guided by the applicant providing statements about their originality, novelty, or significance. |

| | |
|---|---|
| Societal impact | Societal impact refers to the difference research work makes to academia and beyond. Evaluators are frequently asked to examine tangible evidence. Some policies explicitly highlight that societal impact does not equate to publishing in high-impact journals. Forms of societal impact specifically include commercial products, public policies, changing public opinion, or any form of trans-sectoral knowledge transfer. Societal impact relates to a concrete outcome rather than the engagement itself or its reach, which we cover under “Community Service”. |
| Funding | Evidence of a candidate’s ability to acquire competitive external research funding. The concrete evaluation might differ, considering both submitted and successful applications, and looking at their number, perceived prestige of grants or fellowships, or the related income. |
| Farsight | The ability to develop future research directions based on current or anticipated future trends, usually based on a candidate’s narrative on envisaged contributions with regard to research outputs as well as impact on others through mentoring, teaching, or wider service to the profession or communities. |
| Collaborations | Evidence for collaborative work within academia or beyond, specifically covering joint research projects, joint publications, joint supervision of postgraduate students, etc. Such collaborative work could be within the same institution or with external partners. |
| Experience abroad | Looking at whether research experience covers various countries by means of research appointments or visiting positions. This does *not* refer to any short-term visits (< 1 month), e.g. for the purpose of research collaboration or in conjunction with seminars, workshops, or conferences. |
| Presentations | Distinguished from the recognition as invited speaker, this criterion covers the active engagement and dissemination of information by means of technical presentations, science communication displays, or exhibits in meetings such as conferences, seminars, workshops, webinars, or symposia. |
| Professional development | Focuses on the learning for career development, and refers to all forms of continuing professional development, such as attending scholarly meetings (e.g. conferences, symposia, or workshops) or webinars, or participating in dedicated training courses to acquire further skills. |
| Memberships | Non-competitive membership in professional organisations, regardless of any active role. Appointment to committees is not covered under this criterion, but rather under “Service to the profession”. |
| Professional titles | Registered qualifications certified by professional bodies apart from academic degrees, such as Chartered Engineer or Fellow of the Higher Education Academy. |
| Invited positions | All invitations to a professional role, including keynote speaker, session chair, member of an editorial board, elected leadership position in an academy, learned society, or professional body, etc. |
| Awards | Refers to awards, honours and/or formal recognitions received, including election to academies or fellowship of learned societies or professional bodies. |
| Teaching | Summarily covers all teaching-related activities and achievements, the development, delivery, and outcomes. Any teaching-related awards, funding, or publications fall under this rubric, not any of the others. |
| Commercialisation / consultancy | Covers both translating research into commercial applications to generate economic value as well as providing consultancy to industry or public bodies through projects, contracts, and/or appointments. Under this rubric, we refer to the activity, not the outcome. |

| | |
|---|---|
| Service to the profession | Engagement in activities to advance the profession or field of knowledge. This includes peer review of research outputs and funding applications, acting as external examiner, clinical care activities, serving on committees of professional bodies, learned societies, or academies, serving on committees for meetings, or providing any support to foster scholarly communities or build intersectoral relations. |
| Mentoring | All mentoring roles, both in a formal and in an informal capacity. This includes the supervision of students on research projects as well as leadership of teams. Some policies explicitly mention the mentoring of early-career researchers, young undergraduate scholars or junior academic staff. |
| Administrative roles | Administrative roles held by the candidate in service to the institution and/or demonstrated institutional leadership. |
| Community service | The engagement in applying scholarly knowledge and experience to serve the wider community. This covers all forms of science communication, science-related community activities or educational programmes, as well as serving in a technical or advisory role for government or any public bodies. |
| Serving period | The length of time a candidate has served in academia, in a specific role, or at the institution. |
| Interdisciplinarity | Specifies whether the candidate has been involved in interdisciplinary, transdisciplinary or cross-disciplinary collaborative research. |
| Ethics & integrity | Mentioning of ethics or integrity, in particular with respect to publications (e.g. plagiarism), use of animals in research, or involvement of humans in research studies. This does not cover reference to codes of conduct that regulate internal affairs of an institution (such as dealing with students or colleagues). |

#### *1.4.2. Categorisation of criteria*

We found that most policies are explicitly structured around the three broad categories of “research”, “teaching”, and “service”, the latter covering the contributions and impact to the wider profession and society, as well as to the institution and its operations. Moreover, some criteria are frequently used in combination to provide evidence of key traits such as professional standing, accomplishments, or leadership. For further categorisation, we conducted a poll amongst all authors on whether each of our defined criteria resonates with some key trait, with the results shown in Table **S2**. These criteria do not include “interdisciplinarity” and “ethics & integrity”, which are understood to be “general traits” not clearly in the domain of either “research”, “teaching”, or “service”. This led to sub-categories presented in Extended Data Table 3, which best cover all criteria without overlapping. Here 21 criteria related to “research” are grouped into “research outputs” (11), “career development” (8), and “recognition” (2), while 7 criteria relate to “teaching & service”**.** We combined the latter given that “mentoring” was perceived to relate to both.

Some reference to research outputs is found in almost all policies, where research outputs might include publications, registered intellectual property (incl. patents, utility models or registered designs), research data,

research software, as well as creative art works. While there are many possible ways these could be used for assessment, we focus on a restricted number of commonly applied criteria, distinguishing quantitative and qualitative approaches (see Extended Data Table 3), defined as follows: (*1*) Quantitative assessment means evaluation to a number based on data without involving further human judgement and usually relies on scoring and indexing systems (bibliometrics). Quantitative assessment is not necessarily objective, given that bibliometrics themselves can be ultimately based on subjective decisions. (*2*) Qualitative assessment assigns a value to contributions and achievements, usually considering impact and benefits (to the discipline, wider academia, and/or society, depending on context). Sometimes based on narrative descriptions, qualitative assessment is likely to rely on the personal judgement by peer or expert reviewers.

**Table S2. Grouping of assessment criteria into sub-categories.** The heatmap denotes the number of votes by authors who consider the criterion to provide evidence of the trait covered by the prospective sub-category (darker blue implies more votes). The red boxes contain the chosen sub-categories of promotion criteria that best cover all criteria without overlap. The poll was carried out before changing the label "Journal impact factor" to "Journal indexing" and we narrowed down "Research accomplishments" to "Research outputs" as sub-category, as all criteria that resonated with the former fall under the more specific latter.

| | Sub-categories | | | | | | | | | | | | | |
|---|---|---|---|---|---|---|---|---|---|---|---|---|---|---|
| Promotion Criteria | Productivity | Research Accomplishment | Impact of work | Quality of Work | Contribution to the discipline | Innovation / Creativity | Continuous progress in research career | Professional Standing | Leadership | Research strategies for professional growth | Team work | Recognition | Teaching and academic contribution | Services |
| Number of publications | 20 | 19 | 9 | 7 | 15 | 8 | 10 | 11 | 8 | 9 | 9 | 11 | 4 | 1 |
| Recent publications | 20 | 20 | 8 | 8 | 14 | 9 | 20 | 12 | 9 | 14 | 8 | 11 | 5 | 2 |
| Patents | 18 | 19 | 17 | 12 | 14 | 21 | 14 | 11 | 8 | 11 | 3 | 11 | 1 | 1 |
| Citations | 16 | 18 | 19 | 16 | 16 | 7 | 10 | 13 | 8 | 9 | 4 | 12 | 0 | 1 |
| Journal impact factor | 13 | 18 | 16 | 12 | 17 | 10 | 12 | 15 | 10 | 10 | 5 | 15 | 2 | 0 |
| Number of contributing authors | 4 | 7 | 5 | 6 | 5 | 2 | 4 | 3 | 4 | 3 | 18 | 1 | 1 | 0 |
| Authorship order | 13 | 15 | 9 | 7 | 9 | 7 | 5 | 11 | 18 | 7 | 9 | 12 | 1 | 0 |
| Non-metric journal quality | 15 | 17 | 13 | 12 | 17 | 9 | 12 | 15 | 8 | 12 | 5 | 13 | 3 | 0 |
| Role of the authors | 12 | 12 | 9 | 12 | 13 | 11 | 9 | 9 | 11 | 9 | 15 | 11 | 3 | 1 |
| Non-metric quality of publication[illegible] | 9 | 15 | 15 | 19 | 15 | 13 | 11 | 14 | 10 | 9 | 7 | 15 | 3 | 1 |
| Societal Impact of outputs | 10 | 14 | 18 | 17 | 16 | 11 | 11 | 15 | 9 | 10 | 7 | 15 | 3 | 1 |
| Funding | 18 | 15 | 12 | 12 | 10 | 13 | 15 | 15 | 18 | 20 | 14 | 14 | 4 | 6 |
| Farsight | 7 | 6 | 7 | 7 | 9 | 17 | 14 | 8 | 15 | 17 | 7 | 7 | 5 | 6 |
| Collaborations | 13 | 12 | 9 | 8 | 8 | 7 | 8 | 8 | 11 | 17 | 21 | 9 | 3 | 4 |
| Experience abroad | 9 | 13 | 11 | 11 | 10 | 8 | 12 | 15 | 10 | 21 | 12 | 15 | 7 | 6 |
| Presentations | 16 | 12 | 7 | 6 | 9 | 3 | 11 | 8 | 2 | 14 | 4 | 7 | 7 | 1 |
| Professional development | 8 | 8 | 7 | 5 | 10 | 6 | 12 | 9 | 8 | 18 | 5 | 6 | 7 | 3 |
| Membership | 3 | 7 | 5 | 3 | 8 | 2 | 6 | 13 | 7 | 17 | 5 | 9 | 3 | 7 |
| Professional titles | 6 | 5 | 2 | 6 | 6 | 1 | 7 | 14 | 6 | 12 | 2 | 15 | 3 | 5 |
| Invited positions | 11 | 17 | 15 | 12 | 21 | 5 | 12 | 21 | 17 | 13 | 6 | 21 | 6 | 11 |
| Awards | 13 | 15 | 18 | 17 | 20 | 15 | 16 | 21 | 13 | 15 | 7 | 21 | 7 | 7 |
| Commercialisation / consultancy | 10 | 10 | 16 | 8 | 10 | 14 | 10 | 11 | 8 | 9 | 2 | 11 | 2 | 13 |
| Service to the profession | 10 | 6 | 5 | 4 | 13 | 3 | 9 | 12 | 10 | 11 | 9 | 13 | 7 | 18 |
| Mentoring | 11 | 6 | 8 | 7 | 12 | 3 | 8 | 10 | 15 | 10 | 11 | 9 | 15 | 13 |
| Teaching | 7 | 0 | 3 | 3 | 8 | 3 | 5 | 7 | 10 | 5 | 4 | 5 | 21 | 12 |
| Administrative roles | 7 | 0 | 2 | 2 | 1 | 2 | 3 | 11 | 13 | 6 | 7 | 12 | 7 | 19 |
| Community service | 7 | 0 | 10 | 5 | 5 | 4 | 2 | 12 | 13 | 8 | 8 | 14 | 5 | 19 |
| Serving period | 7 | 2 | 4 | 3 | 6 | 2 | 8 | 9 | 7 | 9 | 4 | 9 | 11 | 12 |

### 1.5. Types of documents and their structure

As we report in Extended Data Table 2, documents describing promotion policies were found to be either in the form of a standalone document for Promotion Policy, Guidelines, or Procedures (80%) or embedded within a University or Faculty Guideline or Handbook (20%).[1] The documents varied in structure and level of detail provided. Specifically, for 53% of the policies, we found supporting documentation, such as point scoring systems, rubrics, or evaluation forms (27%), promotion application forms (12%), and/or guidelines for recruitment (13%), while the remaining policies (46%) did not have any supporting documentation and just listed promotion criteria. For 79% of the policies, we identified a section referring exclusively to the role of (full) professor, which specified criteria or scoring that was not shared with other roles. The other policies (28%) included criteria that were exclusive for professors, but also included criteria that were shared with other roles.

Also shown in Extended Data Table 2, the level of detail provided by a policy, we adopted a scale from one to five, with one (1) representing very brief and general policies, and five (5) representing very detailed policies. The scores were defined subjectively, but they were always attributed by the first and last author in order to ensure consistency across documents. As detailed in Table S3, we found that 6% of the policies were brief (scoring less than 3), 22% had a moderate level of detail (scoring 3), and 72% were considered very detailed (scoring higher than 3). A brief policy (score < 3) was typically limited to listing the evaluated criteria, without detailing their definition or the way they should be assessed. A policy with a moderate level of detail (score 3) usually had a defined section (half-page to a page) with criteria exclusive to full professor, describing their definition and a brief mention of the minimum requirements to meet the criteria. Policies scored as detailed (score >3) usually encompassed criteria for different evaluation categories, had discipline or track specific requirements, and had a complete description of how to meet the requirements, potentially including a scoring system, evaluation forms, templates, and other supplementary documentation**.**

For policies that distinguished disciplines and tracks (as explained in sect. 1.2.3-1.2.4 above), we observed variations in both the criteria applied and in how strong candidates need to score on those criteria. We found that slightly more than half of documents included discipline-specific criteria or scores (51%), while a lower proportion of documents surveyed (35%) included track-specific criteria or scores.

Naturally, documents that included discipline-specific scores were often more detailed (82%) than the general ones (63%) [$\chi^2(8) = 40.56$, $p < 0.00001$]. The level of detail, influenced by disciplinary fields, varied, but to a lesser extent. Higher perceptions of completeness were observed for the natural sciences (88%), followed

---

[1] All data in section 1.5 refer to non-weighted observations, because they are meant to represent the composition of the raw data in our sample.

by engineering and technology (88%), social sciences and humanities (82%), and lastly, medicine and health sciences (72%) [$\chi^2(6) = 13.24$, $p = 0.039$] (see Table S3).

A stronger effect was observed between tracks, with policies for the research-focused track perceived to have a higher level of detail (91%), followed by (standard) academic track (75%), clinical (63%), and lastly, teaching-focused (54%) [$\chi^2(8) = 43.58$, $p < 0.000001$]. Furthermore, considering the source of the documents, whether national or institutional (e.g., universities), national policies (78%) were perceived to be slightly more detailed than institutional policies (68%) [$\chi^2(2) = 7.55$, $p = 0.023$] (see Table S3).

**Table S3. Main differences in the sourced documents.** Number of policies for each variable studied, categorised by the level of detail in the structure of the documents as perceived by the data coders. Policies are grouped by tracks, disciplines, the source of the policy, global regions (Global North and Global South), and economic status (income level). The level of detail is represented by scores: < 3 (low detail), = 3 (moderate detail), and > 3 (high detail).

| Level of detail | Score < 3 | Score = 3 | Score >3 |
|---|---|---|---|
| ***Tracks*** | | | |
| Academic | 27 | 79 | 320 |
| Research | 2 | 16 | 185 |
| Teaching | 3 | 15 | 21 |
| Clinical | 1 | 10 | 19 |
| Other | 1 | 2 | 10 |
| ***Disciplines*** | | | |
| Natural Sciences | 0 | 8 | 57 |
| Engineering & technology | 0 | 7 | 49 |
| Medicine & health sciences | 3 | 19 | 56 |
| Social sciences & humanities | 0 | 15 | 68 |
| General | 28 | 71 | 165 |
| ***Source of policy*** | | | |
| National | 12 | 35 | 171 |
| Institutional | 19 | 81 | 214 |
| ***Global North and Global South*** | | | |
| Global North | 11 | 37 | 117 |
| Global South | 20 | 79 | 268 |
| **Economic status (Income level)** | | | |
| High-income | 12 | 45 | 127 |
| Upper-middle income | 8 | 27 | 104 |
| Lower-middle income | 7 | 35 | 87 |
| Low-income | 4 | 9 | 67 |

Conversely, we did not find large differences in the level of detail provided by policies from the Global North and Global South, with a similar number of policies categorised as very detailed: Global North (71%), and Global South (73%) [$\chi^2(2) = 0.40$, $p = 0.818$]. The same was observed by the level of national income, where non-significant differences were observed for each, with low-income countries having 84% of detailed

policies, upper-middle income 75%, lower-middle income 67%, and high income countries 69% [$\chi^2(6) = 9.27$, $p = 0.159$] (Table S3).

### 1.6. Data coding strategy

The coding process was designed to ensure consistency, transparency, and accuracy across all documents. A strict definition of the 30 criteria listed in Table S1, was adopted to guarantee comparability across different contexts. Criteria were scored as present (1) only if they fully adhered to the literal definition; otherwise, they were marked as absent (0). This approach minimised ambiguity and ensured consistent data extraction across all policies.

In addition to the standardised coding template, detailed instruction manuals, working templates, examples, and training videos were developed to guide team members. These resources ensured a uniform understanding of the data coding process. A two-step verification process was also implemented to further improve reliability: in the first step, each policy was tabulated by one team member, then reviewed by at least one other author. Any discrepancies were flagged and resolved with input from the regional team leaders. When necessary, project leaders were consulted to refine criteria definitions or update documentation. This process ensured that the final dataset (a key contribution of our study) was replicable and well-documented.

### 1.7. Weighting - rationale and strategy

Although the dataset can be considered as unbiased with respect to the evaluation criteria that are the object of our study, it is not a random sample of the (unknown) global population of institutions and agencies that set standards and criteria for the evaluation of candidates to full professor. Moreover, as mentioned in section 1.2, some of these institutions and agencies define several policies (i.e. distinct sets of assessment criteria) depending on career track or field of research. These features of the dataset are reflected in a set of post-sampling weights of policies, which we consistently applied to all our analyses. Consequently, weighted averages or proportions in our dataset become indicative of the global fraction of researchers potentially affected, provided that, averaged over many countries, the following assumptions hold: (1) the share of potential candidates for full professorship is proportional to the share of total researchers;[2] (2) the policies set by every institution or agency within a country cover a comparable number of researchers (that is, a comparable number of potential candidates are subject to them); and (3) policies within an institution or

[2] Since the global population of potential candidates for promotion to professor in relation to active researchers is unknown, this assumption is untestable.

agency cover comparable numbers of researchers.[3] Given that we do not aim for our dataset to be representative at the country level or below, these assumptions do not need to hold at the level of specific countries or institutions.

Information on the number of researchers was obtained from country data of the UNESCO world data bank (as of February 2024) using the most recent year for which data are available (typically 2023) [7, 8]. These data however do not cover 23 of the countries included in our dataset, so that we had to rely on other sources or reasonable estimates. For 7 countries (Israel, Bangladesh, Senegal, Sudan, Senegal, Kyrgyz Republic, and Taiwan), we were able to obtain the relevant information from UNESCO reports, SDG hubs, or the Statista website [9–13]. For Comoros, Djibouti, Equatorial Guinea, Guinea-Bissau, Lebanon and Mauritania, the only research data available was the number of publications produced by the country, and this value was used to estimate the number of researchers, using as a reference a country from the region with similar population and GDP per capita. Here, the number of researchers was calculated as a ratio of the reference country, using the number of publications. For 10 countries, the total number of research staff, rather than the number of full-time equivalent (FTE) researchers, is reported. In these cases, we assumed that 50% of the researchers active in the country work full time. As noted by UNESCO (2014 report), the countries for which no data were found are relatively smaller, have lower GDPs and potentially have little to no investment in research. Under these conditions, permanent full-time positions may be scarce, and if any, they will offer low base salaries, and researchers typically engage in other paid activities too [10].[4]

In order to assess the empirical relevance of these assumptions, in Table S4 below we report descriptive statistics for the raw data (unweighted sample), and under alternative assumptions about the share of full-time workers for the countries for which we do not have FTE data. As can be easily seen, weighting has a large impact on the composition of our dataset, e.g. in terms of the proportions of the various categories of analysis that we use in this work. However, the different assumptions about the share of full-time workers (for the countries for which the FTE is not reported) do not have a significant impact because the bulk of uncertainty concerns relatively smaller countries, with low weights in any case. In section 2.5 below we expand on the impact of weighting on our main results.

---

[3] We looked at the distribution of discipline-specific policies over our four discipline groups, wondering whether any of these groups could have substantially more (different) policies on average than others within the same institution. Although the assumption that numbers are quite similar is untestable, this assumption too does not appear to be violated in our sample: the natural sciences have 2.5 policies on average, with a standard deviation $\sigma$ = 2.5; medicine and the health sciences have 1.9 policies on average, with $\sigma$ = 1.5; the social sciences 2.9 with $\sigma$ = 2.7; and engineering 3.1 with $\sigma$ = 4. Accordingly, the use of an equal weight across policies within each institution does not produce different results from those we would obtain if instead we used for example the same weight across policies within each group of disciplines in each institution. Further information is available from the authors upon request.

[4] As no other research data was available for Yemen, the number of active researchers was estimated based on population and GDP, using the average values for the countries for which the information is known.

**Table S4. Sample distribution under different assumptions on the share of full-time staff among all researchers.**

| CLASS | No weight (%) | Full time share | | | |
|---|---|---|---|---|---|
| | | 100% | 50% | 25% | 75% |
| **Global region** | | | | | |
| Global North | 30.9 | 53.2 | 53.2 | 53.3 | 53.2 |
| Global South | 69.1 | 46.8 | 46.8 | 46.7 | 46.8 |
| **Continent** | | | | | |
| Africa | 38.8 | 3.10 | 3.20 | 3.10 | 3.20 |
| Asia | 22.2 | 46.30 | 46.30 | 46.4 | 46.30 |
| Europe | 16.4 | 27.60 | 27.60 | 27.7 | 27.60 |
| Latin America | 7.70 | 3.60 | 3.70 | 3.60 | 3.60 |
| North America | 10.7 | 18.00 | 18.00 | 18.0 | 18.00 |
| Oceania | 4.10 | 1.30 | 1.30 | 1.30 | 1.30 |
| **Policy scope** | | | | | |
| Institutional policies | 58.90 | 83.90 | 83.80 | 84.00 | 83.80 |
| National policies | 41.10 | 16.10 | 16.20 | 16.00 | 16.20 |
| **Income level** | | | | | |
| High-income | 34.50 | 51.70 | 51.60 | 51.80 | 51.70 |
| Upper-middle income | 26.20 | 38.80 | 38.80 | 38.80 | 38.80 |
| Lower-middle income | 24.30 | 9.20 | 9.20 | 9.20 | 9.20 |
| Low-income | 15.10 | 0.30 | 0.30 | 0.30 | 0.30 |
| **Disciplines** | | | | | |
| General | 48.80 | 68.40 | 68.40 | 68.30 | 68.40 |
| Natural sciences | 10.90 | 3.80 | 3.80 | 3.80 | 3.80 |
| Medical & Health sciences | 14.10 | 7.40 | 7.40 | 7.40 | 7.40 |
| Social sciences & Humanities | 15.60 | 8.30 | 8.30 | 8.30 | 8.30 |
| Engineering & technology | 10.50 | 12.10 | 12.10 | 12.10 | 12.10 |
| **Track** | | | | | |
| Academic track | 52.00 | 80.30 | 80.20 | 80.30 | 80.20 |
| Research track | 34.50 | 6.10 | 6.10 | 6.00 | 6.10 |
| Teaching track | 6.00 | 6.10 | 6.10 | 6.10 | 6.10 |
| Clinical or other | 7.50 | 7.60 | 7.60 | 7.60 | 7.60 |

## 2. ADDITIONAL RESULTS

### 2.1. Average differences in factor scores across policy categories

We performed Wald tests to identify systematic differences in factor scores among the different policy categories, namely, policies implemented by single institutions or nationwide, policies implemented in the Global North or the Global South, policies specific to each discipline, and policies categorised by the economic status of the country. All tests use post-sampling weights.

As shown in Extended Data Table 5 and Extended Data Fig 4, the factors' means do not appear to significantly differ between national and institutional policies; they do for all factors except for career development, when distinguishing between Global North and South, and between countries of different economic statuses; they differ across disciplines, except for visibility and engagement; and among tracks they differ in the cases of output metrics and visibility and engagement. However, we stress that these differences could arise from spurious correlations and indeed, the results of the later regression analysis show that not all of them remain statistically significant, once controlling for other factors (see Table S7 further below). For example, while Extended Data Figure 4 shows a higher adoption of output metrics by the Global South, it is clear from Figure 5 and Table S7 (below) that this is driven by the economic status of a country. These analyses nonetheless show that families of criteria, as defined by the four factors, span a large variety of cases, and that policies are more diversified than is frequently thought. Moreover, even if this can be explained by other variables, the fact remains that potential candidates face certain policies that often are different, on average, across categories (e.g., between Global North and Global South).

### 2.2. Proportion of the single criteria across categories

We conducted Pearson chi-squared ($\chi^2$) tests to examine systematic differences in individual assessment criteria across different policy categories on the weighted data. These tests measure the difference between the observed and expected frequencies under the assumption of independence between the variables. The results of the tests are below, in Table S5.

**Table S5. Systematic differences in individual assessment criteria.** Pearson $\chi^2$ tests were performed to assess differences in the distributions of criteria within the different comparative analyses performed in this study: scope of policies (national vs institutional), regional (Global North vs South), country economic status (high, upper-middle, lower-middle, and low-income countries), and analysis by discipline. The null hypothesis ($H_0$) assumes no differences (diff = 0) in the distributions of the criteria; $\chi^2$ = Pearson chi-squared statistics with the degrees of freedom shown in parenthesis; $F$ = design-based $F$-statistic (i.e., accounting for post-sampling weights) with degrees of freedom shown in parenthesis. $\chi^2$ and values highlighted in yellow are at least 5 times higher than the critical value at a 0.05% significance level, while values lower than the critical value have grey font. Similarly, $F$ test values in yellow and bold, are at least 2x > critical value. In both cases, values in bold are above the 0.05% significance level, while values in grey are below it. $P$ = confidence level in terms of $p$-value. p-values < 0.01 are highlighted in dark green and in white font, p-values between 0.01- 0.05 are highlighted in light green and with bold font, and p-values > 0.05 have grey font.

| CRITERIA | Institutional vs. national policies | | | Global North vs. Global South | | | Economic status | | | | Disciplines | | | | Tracks | | | |
|---|---|---|---|---|---|---|---|---|---|---|---|---|---|---|---|---|---|---|
| | χ2 (1) | Design-based: F(1, 531) | P | χ2(1) | Design-based: F(1, 531) | P | χ2 (3) | Design-based | F | P | χ2 (4) | Design-based | F | P | χ2 (3) | Design-based | F | P |
| N. publications | 8.128 | 1.354 | 0.246 | 26.557 | 2.080 | 0.150 | 167.035 | F(1.80, 479.96) | 16.335 | 0.000 | 77.196 | F(2.39, 640.21) | 11.544 | 0.000 | 16.159 | F(2.57, 687.96) | 1.259 | 0.287 |
| No. publications | 23.016 | 4.065 | 0.045 | 22.041 | 2.862 | 0.092 | 52.639 | F(1.47, 391.66) | 3.593 | 0.042 | 29.382 | F(3.24, 869.43) | 1.939 | 0.117 | 6.101 | F(2.69, 721.70) | 0.490 | 0.669 |
| Patents | 4.266 | 0.985 | 0.322 | 2.901 | 0.246 | 0.620 | 20.676 | F(1.69, 451.61) | 1.877 | 0.161 | 65.209 | F(3.44, 922.87) | 5.341 | 0.001 | 30.523 | F(2.49, 668.27) | 3.124 | 0.034 |
| Citations | 1.491 | 0.258 | 0.612 | 212.442 | 18.641 | 0.000 | 2.649 | F(1.46, 389.00) | 0.123 | 0.819 | 14.006 | F(2.99, 800.84) | 1.087 | 0.354 | 17.915 | F(2.43, 651.44) | 1.668 | 0.182 |
| Journal indexing | 41.052 | 6.059 | 0.015 | 27.259 | 1.505 | 0.221 | 149.919 | F(1.36, 362.08) | 9.297 | 0.001 | 144.487 | F(2.95, 789.84) | 13.319 | 0.000 | 20.431 | F(2.16, 579.95) | 2.131 | 0.115 |
| No. of authors | 0.506 | 0.081 | 0.776 | 101.903 | 11.166 | 0.001 | 35.090 | F(1.56, 417.32) | 2.352 | 0.109 | 12.418 | F(2.52, 674.86) | 0.962 | 0.399 | 12.495 | F(2.51, 673.38) | 2.296 | 0.088 |
| Authorship order | 10.842 | 1.824 | 0.178 | 70.430 | 10.680 | 0.001 | 111.352 | F(1.79, 478.33) | 9.765 | 0.000 | 55.743 | F(3.01, 807.43) | 3.067 | 0.027 | 19.248 | F(2.37, 635.72) | 1.878 | 0.146 |
| Non-metric Journal quality | 1.201 | 0.196 | 0.659 | 15.855 | 1.380 | 0.241 | 42.482 | F(1.59, 423.81) | 3.801 | 0.032 | 32.397 | F(2.42, 649.57) | 3.766 | 0.017 | 5.121 | F(2.51, 671.92) | 0.452 | 0.681 |
| Role of authors | 4.476 | 0.763 | 0.383 | 127.007 | 37.267 | 0.000 | 39.879 | F(1.07, 285.92) | 2.950 | 0.084 | 23.342 | F(2.93, 784.85) | 2.219 | 0.086 | 70.560 | F(2.59, 695.23) | 6.026 | 0.001 |
| Non-metric publications quality | 32.117 | 19.622 | 0.000 | 0.171 | 0.013 | 0.909 | 164.186 | F(1.91, 510.74) | 32.803 | 0.000 | 49.767 | F(2.56, 687.23) | 7.993 | 0.000 | 3.143 | F(2.39, 640.79) | 0.297 | 0.782 |
| Social impact | 7.609 | 1.380 | 0.241 | 0.142 | 0.008 | 0.927 | 17.358 | F(1.58, 421.39) | 0.954 | 0.368 | 26.297 | F(1.91, 511.89) | 1.032 | 0.354 | 9.373 | F(2.29, 614.49) | 0.776 | 0.477 |
| Farsight | 50.596 | 36.016 | 0.000 | 4.166 | 0.713 | 0.399 | 34.534 | F(1.06, 281.95) | 1.978 | 0.160 | 59.965 | F(2.37, 635.24) | 3.073 | 0.038 | 15.398 | F(2.72, 729.09) | 1.374 | 0.251 |
| Funding | 9.805 | 1.968 | 0.162 | 9.416 | 0.625 | 0.430 | 1.532 | F(1.74, 464.25) | 0.148 | 0.834 | 32.507 | F(3.20, 857.96) | 3.318 | 0.017 | 40.164 | F(2.21, 591.01) | 4.324 | 0.011 |
| Collaborations | 11.280 | 2.619 | 0.107 | 0.030 | 0.002 | 0.963 | 36.771 | F(1.36, 362.24) | 1.678 | 0.196 | 25.676 | F(1.82, 487.56) | 1.032 | 0.352 | 2.641 | F(2.14, 573.22) | 0.222 | 0.816 |
| Experience abroad | 0.491 | 0.103 | 0.749 | 20.248 | 2.233 | 0.136 | 1.804 | F(1.37, 367.10) | 0.083 | 0.850 | 9.718 | F(2.60, 697.34) | 0.719 | 0.522 | 7.494 | F(2.14, 572.26) | 1.216 | 0.299 |
| Presentations | 14.154 | 2.573 | 0.110 | 8.969 | 0.810 | 0.369 | 42.273 | F(1.73, 461.25) | 3.180 | 0.050 | 17.652 | F(3.26, 873.68) | 1.085 | 0.357 | 8.192 | F(2.40, 644.14) | 0.681 | 0.533 |
| Professional development | 0.127 | 0.025 | 0.874 | 10.551 | 1.538 | 0.216 | 3.615 | F(1.48, 395.20) | 0.197 | 0.753 | 20.715 | F(3.20, 858.43) | 1.644 | 0.174 | 6.644 | F(2.19, 586.96) | 0.677 | 0.521 |
| Memberships | 0.832 | 0.175 | 0.676 | 17.299 | 9.240 | 0.003 | 27.965 | F(1.94, 518.12) | 2.770 | 0.065 | 14.806 | F(2.11, 564.46) | 1.855 | 0.155 | 12.446 | F(2.44, 654.25) | 1.079 | 0.350 |
| Professional Titles | 5.284 | 13.549 | 0.000 | 92.374 | 10.140 | 0.002 | 32.097 | F(1.86, 496.65) | 50.037 | 0.000 | 19.722 | F(1.77, 474.22) | 2.226 | 0.116 | 10.998 | F(1.19, 319.72) | 5.268 | 0.017 |
| Invited positions | 2.861 | 0.492 | 0.484 | 10.302 | 1.199 | 0.275 | 182.396 | F(1.61, 429.79) | 17.432 | 0.000 | 45.091 | F(2.83, 758.80) | 2.408 | 0.070 | 36.389 | F(1.60, 428.21) | 3.294 | 0.049 |
| Awards | 6.683 | 1.162 | 0.282 | 8.235 | 0.564 | 0.453 | 12.999 | F(1.66, 443.97) | 0.949 | 0.374 | 25.042 | F(2.83, 758.30) | 1.429 | 0.235 | 19.654 | F(2.14, 574.39) | 2.234 | 0.104 |
| Commercialisation/Consultancy | 0.154 | 0.030 | 0.862 | 73.046 | 5.217 | 0.023 | 0.643 | F(1.42, 378.42) | 0.029 | 0.931 | 39.755 | F(1.63, 435.88) | 1.761 | 0.180 | 0.259 | F(2.13, 571.61) | 0.023 | 0.982 |
| Service to the profession | 14.392 | 2.408 | 0.122 | 3.382 | 0.237 | 0.627 | 129.853 | F(1.50, 400.42) | 7.778 | 0.002 | 72.042 | F(2.76, 738.45) | 4.638 | 0.004 | 6.972 | F(1.96, 525.57) | 0.483 | 0.614 |
| Mentoring | 2.157 | 0.288 | 0.592 | 7.504 | 1.977 | 0.161 | 12.601 | F(1.43, 382.93) | 0.800 | 0.413 | 52.544 | F(2.03, 544.64) | 2.067 | 0.127 | 17.592 | F(2.38, 638.91) | 1.320 | 0.268 |
| Teaching | 0.880 | 0.305 | 0.581 | 33.822 | 2.303 | 0.130 | 10.143 | F(1.76, 469.16) | 1.319 | 0.267 | 1.348 | F(2.40, 643.91) | 0.217 | 0.843 | 161.460 | F(2.63, 704.30) | 13.126 | 0.000 |
| Administrative roles | 0.128 | 0.021 | 0.885 | 0.151 | 0.011 | 0.916 | 114.025 | F(1.56, 416.51) | 6.624 | 0.004 | 14.009 | F(2.06, 551.14) | 0.471 | 0.630 | 11.852 | F(2.07, 553.59) | 0.792 | 0.457 |
| Community service | 18.005 | 2.988 | 0.085 | 13.558 | 0.877 | 0.350 | 7.608 | F(1.53, 409.49) | 0.374 | 0.632 | 18.201 | F(2.19, 588.13) | 0.618 | 0.554 | 9.466 | F(2.33, 624.74) | 0.746 | 0.494 |
| Serving period | 15.095 | 3.718 | 0.055 | 81.904 | 27.660 | 0.000 | 29.974 | F(1.33, 356.39) | 1.271 | 0.272 | 13.990 | F(2.77, 742.73) | 0.985 | 0.395 | 42.097 | F(2.23, 598.59) | 3.581 | 0.024 |
| Interdisciplinarity | 14.526 | 4.818 | 0.029 | 91.374 | 9.016 | 0.003 | 103.301 | F(1.29, 345.29) | 27.270 | 0.000 | 21.518 | F(2.20, 589.26) | 3.129 | 0.040 | 2.161 | F(2.17, 580.70) | 0.262 | 0.787 |
| Ethics & integrity | 0.378 | 0.058 | 0.810 | 91.374 | 12.119 | 0.001 | 70.063 | F(1.54, 411.33) | 3.970 | 0.029 | 97.565 | F(2.27, 609.53) | 5.308 | 0.004 | 15.398 | F(2.16, 579.64) | 1.151 | 0.320 |

## 2.3. Power of the analysis

As is well known, the power of statistical tests depends on sample size, variability of the relevant variable(s), and effect size. The reference population of potential candidates for full professorship globally is unknown and unobservable, therefore we consider here the variability of the evaluation criteria and the differences in proportions or mean values across categories, as observed in our own sample. To assess the power of the various tests, we set a reference statistical significance level for the difference in proportions or in means ($\alpha$) at 5%, we allow for the sizes and the variances of the two groups or categories to be different, and we use post-sampling weights.

We consider two examples: for proportions, the first of the evaluation criteria considered, i.e. the candidate's number of publications; and for mean values, the first of our factors, i.e. output metrics.

In the main text we report on several differences in proportions or means between different aggregates of national and/or institutional policies. Such analyses can be considered at different levels, implying comparisons of different groups of countries, institutions/agencies, or policies. We consider all these cases in Table S6, where we report the estimated power of tests on the difference in proportions or means between (*1*) countries and academic institutions; and between Global North and Global South, separately among: (*2*) national policies; (*3*) institutional policies, considering the number of different institutions; and (*4*) institutional policies, considering the number of policies. Figure S1 reports the estimated power for the same tests, as a function of sample size.

As can be seen, for all tests the estimated power is above 0.95, except for the tests involving the number of different countries, when it is above 0.9. In this latter case, however, we notice that the estimation is of lower relevance because there is not a large population of countries from which to randomly sample a certain dataset of countries. Indeed, our dataset of 121 countries comprises half of the countries and territories in the planet, including all the most populous ones, and therefore it constitutes a very large fraction of the whole set (population) of countries. In any case, we do not run any analyses at the country level and both in the main manuscript and in this Supplementary Information we only consider policies.

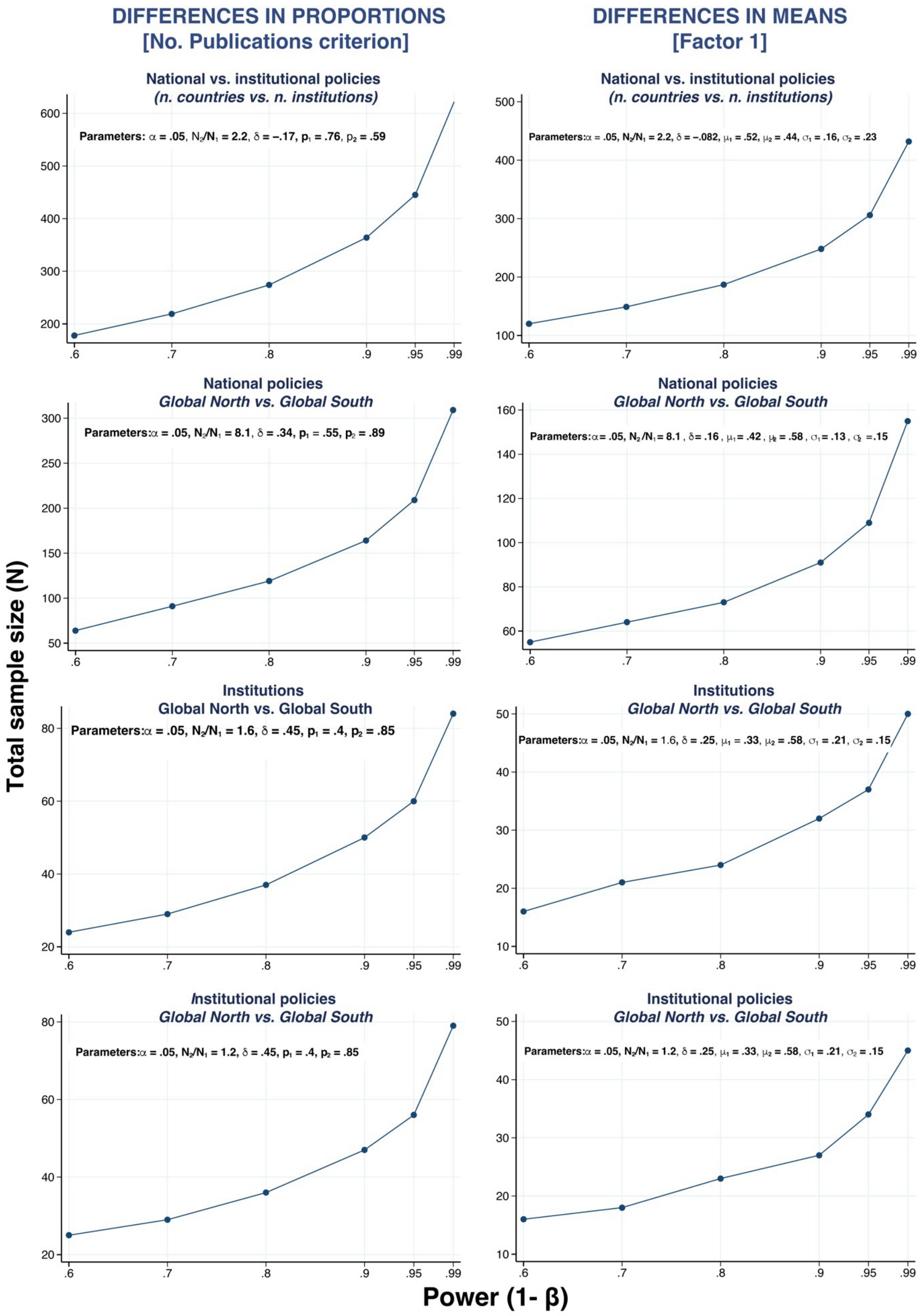


**Figure S1. Estimated power as a function of sample size,** for the tests reported in Table S6. In our dataset, $n = 390$ for countries vs. institutions; $n = 121$ for national policies; $n = 190$ for institutions or agencies; and $n = 314$ for institutional policies.

**Table S6. Estimated power of the statistical tests.** This table presents the estimates of statistical tests for the differences in proportions (for candidates' number of publications) or in means (for the factor Output metrics), for several tests shown in the main manuscript. The column "Proportions" reports the estimated power for a two-sample proportions test *(Pearson's chi-squared test)* of $H_0$: $p_2 = p_1$ *versus* $H_a$: $p_2 != p_1$. The column *"Means"* reports the estimated power for a two-sample means t-test assuming unequal variance*s of* $H_0$: $m_2 = m_1$ *versus* $H_a$: $m_2 != m_1$.

| Values | Countries vs. Institutions | | National policies: Global North / South | | Institutions: Global North/ South | | Policies within institutions Global North / South | |
|---|---|---|---|---|---|---|---|---|
| | Proportions | Means | Proportions | Means | Proportions | Means | Proportions | Means |
| Alpha | 0.05 | 0.05 | 0.05 | 0.05 | 0.05 | 0.05 | 0.05 | 0.05 |
| n | 390 | 390 | 121 | 121 | 190 | 190 | 314 | 314 |
| *$n_1$* | *121* | *121* | *24* | *24* | *73* | *73* | *141* | *141* |
| *$n_2$* | *269* | *269* | *194* | *194* | *117* | *117* | *173* | *173* |
| n2/n1 | 2.220 | 2.220 | 2.780 | 2.780 | 8.080 | 8.080 | 1.230 | 1.230 |
| Difference | -0.173 | -0.082 | 0.335 | 0.158 | 0.446 | 0.250 | 0.446 | 0.250 |
| Proportion 1 | 0.758 | - | 0.551 | - | 0.400 | - | 0.400 | - |
| Proportion 2 | 0.585 | - | 0.887 | - | 0.846 | - | 0.846 | - |
| Mean 1 | - | 0.519 | - | 0.422 | - | 0.333 | - | 0.333 |
| Mean 2 | - | 0.437 | - | 0.580 | - | 0.583 | - | 0.583 |
| Std. Dev. 1 | - | 0.160 | - | 0.131 | - | 0.211 | - | 0.211 |
| Std. Dev. 2 | - | 0.225 | - | 0.151 | - | 0.154 | - | 0.154 |

## 2.4. Regression coefficients for full sample analysis and supporting statistical analyses

We report in Table S7 the full results of the regressions of the four factors on the explanatory variables explored in this study. As explained in the text, these regressions are provided for the sake of multivariate descriptive analysis, to consider the conditional correlation between the factors and a number of variables, with no causal interpretation.

**Table S7. Regression results from the 121 analysed countries.** This table presents the full regressions results for the four factors, using two alternative models: **[1]** includes "Global South" and "National policies" as explanatory variables (with reference categories: "Global North" and "Institutional policies" respectively); **[2]** includes the combined variables "National Policies in the Global South", "National Policies in the Global North", and "Institutional Policies in the Global South" (reference category is "Institutional Policies in the Global North"). For each variable, the following is provided**:** Regression coefficients (Row 1, **bold**); Heteroskedasticity-robust standard errors clustered by policy document (Row 2, grey font, in brackets); Two-sided *t*-tests of difference from zero (Row 3, blue font); *p*-values implied by the t-tests (Row 4, orange font). * next to the coefficients denotes statistical significance in terms of *p* values, where ***: $p < 0.01$, **: $p < 0.05$, and *: $p < 0.1$.

| CLASSES | Factor 1<br>Output metrics | | Factor 2<br>Visibility, engagement | | Factor 3<br>Career development | | Factor 4<br>Outcomes & impact | |
|---|---|---|---|---|---|---|---|---|
| | [1] | [2] | [1] | [2] | [1] | [2] | [1] | [2] |
| *Global Region and Policy Scope (Independent)* | | | | | | | | |
| National policies | **0.0699*** ** | | **-0.0128** | | **0.0464** | | **-0.0636** | |
| | [0.0256] | | [0.0589] | | [0.0441] | | [0.0669] | |
| | 2.7260 | | -0.2170 | | 1.0540 | | -0.9520 | |
| | 0.0068 | | 0.8280 | | 0.2930 | | 0.3420 | |
| Global South | **0.219*** ** | | **0.211*** ** | | **0.114** ** | | **0.0464** | |
| | [0.0455] | | [0.0535] | | [0.0574] | | [0.1020] | |
| | 4.8030 | | 3.9400 | | 1.9900 | | 0.4550 | |
| | 0.0000 | | 0.0001 | | 0.0476 | | 0.6490 | |
| *Global Region and Policy Scope (clustered)* | | | | | | | | |
| National policies, Global North | | **0.101*** ** | | **-0.122* ** | | **0.0198** | | **-0.144** |
| | | [0.0350] | | [0.0625] | | [0.0559] | | [0.1120] |
| | | 2.8820 | | -1.9590 | | 0.3540 | | -1.2850 |
| | | 0.0043 | | 0.0512 | | 0.7240 | | 0.2000 |
| Institutional policies, Global South | | **0.223*** ** | | **0.193*** ** | | **0.110* ** | | **0.0336** |
| | | [0.0462] | | [0.0506] | | [0.0598] | | [0.1000] |
| | | 4.8400 | | 3.8190 | | 1.8420 | | 0.3350 |
| | | 0.0000 | | 0.0002 | | 0.0666 | | 0.7380 |
| National policies, Global South | | **0.263*** ** | | **0.289*** ** | | **0.183*** ** | | **0.0491** |
| | | [0.0558] | | [0.0785] | | [0.0680] | | [0.1050] |
| | | 4.7120 | | 3.6800 | | 2.6880 | | 0.4660 |
| | | 0.0000 | | 0.0003 | | 0.0076 | | 0.6410 |
| *Disciplines* | | | | | | | | |
| Natural sciences | **0.0251** | **0.0259** | **-0.0349** | **-0.0377** | **0.000813** | **0.000111** | **0.00250** | **0.000384** |
| | [0.0480] | [0.0463] | [0.0438] | [0.0472] | [0.0363] | [0.0372] | [0.0565] | [0.0598] |
| | 0.5220 | 0.5590 | -0.7960 | -0.8000 | 0.0224 | 0.0030 | 0.0442 | 0.0064 |
| | 0.6020 | 0.5760 | 0.4270 | 0.4250 | 0.9820 | 0.9980 | 0.9650 | 0.9950 |
| Medicine & health sciences | **0.0195** | **0.0170** | **-0.0582* ** | **-0.0495** | **0.00665** | **0.00875** | **-0.0397** | **-0.0334** |
| | [0.0392] | [0.0404] | [0.0328] | [0.0332] | [0.0442] | [0.0439] | [0.0515] | [0.0528] |
| | 0.4970 | 0.4220 | -1.7760 | -1.4900 | 0.1510 | 0.2000 | -0.7710 | -0.6330 |
| | 0.6200 | 0.6740 | 0.0769 | 0.1370 | 0.8800 | 0.8420 | 0.4410 | 0.5270 |
| Social sciences & humanities | **0.00761** | **0.00887** | **-0.0173** | **-0.0218** | **-0.0579** | **-0.0590** | **0.0156** | **0.0124** |
| | [0.0340] | [0.0336] | [0.0384] | [0.0328] | [0.0670] | [0.0652] | [0.0462] | [0.0454] |
| | 0.2240 | 0.2640 | -0.4500 | -0.6650 | -0.8640 | -0.9040 | 0.3390 | 0.2720 |
| | 0.8230 | 0.7920 | 0.6530 | 0.5070 | 0.3880 | 0.3670 | 0.7350 | 0.7860 |
| Engineering & technology | **0.0322** | **0.0286** | **-0.0127** | **0.000321** | **-0.145* ** | **-0.142* ** | **0.164*** ** | **0.174*** ** |
| | [0.0396] | [0.0403] | [0.0445] | [0.0423] | [0.0814] | [0.0838] | [0.0526] | [0.0541] |
| | 0.8150 | 0.7080 | -0.2850 | 0.0076 | -1.7860 | -1.6960 | 3.1210 | 3.2120 |
| | 0.4160 | 0.4790 | 0.7760 | 0.9940 | 0.0753 | 0.0910 | 0.0020 | 0.0015 |
| *Tracks* | | | | | | | | |
| Research track | **-0.0271** | **-0.0214** | **0.147*** ** | **0.127*** ** | **-0.201** ** | **-0.206** ** | **0.109** ** | **0.0937** ** |
| | [0.0287] | [0.0279] | [0.0380] | [0.0453] | [0.0818] | [0.0811] | [0.0425] | [0.0449] |
| | -0.9450 | -0.7660 | 3.8710 | 2.7970 | -2.4560 | -2.5370 | 2.5540 | 2.0860 |
| | 0.3450 | 0.4440 | 0.000136 | 0.00553 | 0.0147 | 0.0117 | 0.0112 | 0.0379 |
| Teaching track | -0.00374 | -0.00201 | -0.0135 | -0.0196 | -0.0356 | -0.0371 | -0.162 | -0.166 |
| | [0.0551] | [0.0557] | [0.0661] | [0.0645] | [0.0510] | [0.0504] | [0.116] | [0.113] |
| | -0.0678 | -0.0361 | -0.205 | -0.304 | -0.698 | -0.736 | -1.395 | -1.477 |
| | 0.9460 | 0.9710 | 0.8380 | 0.7610 | 0.4860 | 0.4620 | 0.1640 | 0.1410 |
| Clinical or other | 0.0207 | 0.0242 | -0.0182 | -0.0303 | -0.00531 | -0.00825 | -0.0906 | -0.0994* |
| | [0.0462] | [0.0465] | [0.0396] | [0.0389] | [0.0618] | [0.0620] | [0.0559] | [0.0548] |
| | 0.4490 | 0.5200 | -0.4600 | -0.7790 | -0.0859 | -0.1330 | -1.6190 | -1.8150 |
| | 0.6540 | 0.6030 | 0.6460 | 0.4370 | 0.9320 | 0.8940 | 0.1070 | 0.0706 |

| | | | | | | | | |
|---|---|---|---|---|---|---|---|---|
| ***Continents*** | | | | | | | | |
| Africa | **-0.0464** | **-0.0424** | **-0.0957** | **-0.110** | **-0.0470** | **-0.0504** | **0.172** | **0.162** |
| | [0.0707] | [0.0739] | [0.0846] | [0.0758] | [0.0605] | [0.0590] | [0.125] | [0.116] |
| | -0.6550 | -0.5740 | -1.1300 | -1.4460 | -0.7780 | -0.8550 | 1.3800 | 1.3990 |
| | *0.5130* | *0.5660* | *0.25900* | *0.1490* | *0.4370* | *0.3930* | *0.1690* | *0.1630* |
| Asia | **-0.0562** | **-0.0494** | **-0.177***** | **-0.201***** | **-0.157***** | **-0.163***** | **0.143**** | **0.126**** |
| | [0.0351] | [0.0367] | [0.0600] | [0.0596] | [0.0384] | [0.0405] | [0.0621] | [0.0631] |
| | -1.6010 | -1.3480 | -2.9410 | -3.3630 | -4.0940 | -4.0200 | 2.3100 | 1.9940 |
| | *0.1100* | *0.1790* | *0.00356* | *0.00088* | *5.63E-05* | *7.59E-05* | *0.0217* | *0.0471* |
| Latin America | **-0.303***** | **-0.297***** | **-0.203***** | **-0.224***** | **-0.0854** | **-0.0904** | **0.156*** | **0.141** |
| | [0.0688] | [0.0686] | [0.0677] | [0.0711] | [0.0905] | [0.0866] | [0.0862] | [0.0892] |
| | -4.404 | -4.328 | -2.998 | -3.149 | -0.944 | -1.044 | 1.813 | 1.581 |
| | *1.54E-05* | *2.13E-05* | *0.0030* | *0.0018* | *0.3460* | *0.2980* | *0.0710* | *0.1150* |
| North America | **0.0778*** | **0.0845*** | **0.111*** | **0.0869** | **-0.0355** | **-0.0413** | **0.0215** | **0.00409** |
| | [0.0457] | [0.0464] | [0.0606] | [0.0609] | [0.0484] | [0.0497] | [0.0711] | [0.0743] |
| | 1.7030 | 1.8200 | 1.8240 | 1.4260 | -0.7340 | -0.8300 | 0.3020 | 0.0550 |
| | *0.0897* | *0.0699* | *0.0693* | *0.155* | *0.463* | *0.407* | *0.763* | *0.956* |
| Oceania | **-0.0541** | **-0.0481** | **0.00245** | **-0.0187** | **0.0133** | **0.00812** | **0.217***** | **0.202***** |
| | [0.0524] | [0.0531] | [0.0736] | [0.0733] | [0.0672] | [0.0679] | [0.0691] | [0.0713] |
| | -1.0320 | -0.9070 | 0.0333 | -0.2560 | 0.1970 | 0.120 | 3.1450 | 2.8290 |
| | *0.3030* | *0.3650* | *0.9730* | *0.7980* | *0.8440* | *0.9050* | *0.0019* | *0.0051* |
| ***Economic Status (Income level)*** | | | | | | | | |
| Upper-middle income | **0.124***** | **0.123***** | **-0.149***** | **-0.148***** | **0.0178** | **0.0181** | **-0.145** | **-0.144** |
| | [0.0341] | [0.0340] | [0.0467] | [0.0460] | [0.0533] | [0.0535] | [0.0974] | [0.0956] |
| | 3.6320 | 3.6270 | -3.1940 | -3.2110 | 0.3330 | 0.3390 | -1.4880 | -1.5050 |
| | *0.0003* | *0.0003* | *0.0016* | *0.0015* | *0.7390* | *0.7350* | *0.1380* | *0.1340* |
| Lower-middle income | **0.00916** | **0.0261** | **-0.137**** | **-0.197***** | **-0.00268** | **-0.0172** | **-0.140** | **-0.183*** |
| | [0.0439] | [0.0468] | [0.0639] | [0.0660] | [0.0578] | [0.0602] | [0.113] | [0.105] |
| | 0.2090 | 0.5580 | -2.1520 | -2.9920 | -0.0463 | -0.2870 | -1.2340 | -1.7410 |
| | *0.8350* | *0.5770* | *0.0323* | *0.0030* | *0.9630* | *0.7750* | *0.2180* | *0.0828* |
| Low-income | **-0.0455** | **-0.0323** | **-0.196**** | **-0.243***** | **-0.0189** | **-0.0302** | **-0.328**** | **-0.362***** |
| | [0.0720] | [0.0761] | [0.0797] | [0.0792] | [0.0710] | [0.0718] | [0.142] | [0.133] |
| | -0.6320 | -0.4240 | -2.4620 | -3.0670 | -0.2660 | -0.4210 | -2.3090 | -2.7320 |
| | *0.5280* | *0.6720* | *0.0144* | *0.0024* | *0.7900* | *0.6740* | *0.0217* | *0.0067* |
| **Criteria specific for full professor** | | | | | | | | |
| Policy specific for full prof. | **-0.0393** | **-0.0387** | **0.0471** | **0.0451** | **-0.191**** | **-0.191**** | **-0.00703** | **-0.00853** |
| | [0.0436] | [0.0428] | [0.0330] | [0.0325] | [0.0780] | [0.0784] | [0.0584] | [0.0603] |
| | -0.9020 | -0.9040 | 1.4290 | 1.3890 | -2.4440 | -2.4370 | -0.1200 | -0.1410 |
| | *0.3680* | *0.3670* | *0.1540* | *0.1660* | *0.0152* | *0.0155* | *0.9040* | *0.8880* |
| ***Regression Parameters*** | | | | | | | | |
| Constant | **0.362***** | **0.354***** | **0.326***** | **0.354***** | **0.670***** | **0.677***** | **0.620***** | **0.641***** |
| | [0.0478] | [0.0479] | [0.0550] | [0.0572] | [0.0718] | [0.0720] | [0.0831] | [0.0896] |
| | 7.5880 | 7.4000 | 5.9160 | 6.1920 | 9.3310 | 9.3930 | 7.4540 | 7.1480 |
| | *0.0000* | *0.0000* | *0.0000* | *0.0000* | *0.0000* | *0.0000* | *0.0000* | *0.0000* |
| Observations | 532 | 532 | 532 | 532 | 532 | 532 | 532 | 532 |
| R-squared | 0.569 | 0.389 | 0.572 | 0.391 | 0.356 | 0.264 | 0.399 | 0.277 |

**Table S8. Statistics for the baseline and alternative regressions.** For each dependent variable (factor), the table shows the information criteria statistics for a regression containing only characteristics of the policy document (continent; global region; and economic status of the country) as explanatory variables in the "no job characteristics" specification; for one containing both characteristics of the policy document and job characteristics (disciplinary field; track; and whether the policy is specific for full professor) in the "full model" specification; for the full model excluding the disciplinary field only, in the "excluding discipline" specification; and for the same sets of variables but disaggregating the policy kind (institutional and national) by global regions, in the specifications denoted by "disaggregated inst./geo.". N = observations; ll(null) = log-likelihood of a constant-only model; ll(model) = log-likelihood of the model; df = degrees of freedom; AIC = Akaike information criterion; and BIC = Bayesian information criterion (BIC). Bold chars denote the preferred specification.

| Policy Features | N | ll(null) | ll(model) | df | AIC | BIC |
|---|---|---|---|---|---|---|
| **Factor 1: Output metrics** | | | | | | |
| No job characteristics | 531 | 170.2 | 387.2 | 11 | -752.3 | -705.3 |
| Excluding discipline | 531 | 170.2 | 391.7 | 15 | -753.4 | -689.3 |
| Full model specification | 531 | 170.2 | 393.8 | 19 | -749.6 | -668.3 |
| No job chars; disaggregated inst./geo. | 531 | 170.2 | 389.6 | 12 | -755.2 | -703.9 |
| Excluding discipline; disaggregated inst./geo. | 531 | 170.2 | 393.9 | 16 | -755.8 | -687.4 |
| Full model; disaggregated inst./geo. | 531 | 170.2 | 395.6 | 20 | -751.2 | -665.7 |
| **Factor 2: Visibility and engagement** | | | | | | |
| No job characteristics | 531 | 212.3 | 302.8 | 11 | -583.5 | -536.5 |
| Excluding discipline | 531 | 212.3 | 325.6 | 15 | -621.2 | -557.1 |
| Full model specification | 531 | 212.3 | 329.0 | 19 | -620.1 | -538.8 |
| No job chars; disaggregated inst./geo. | 531 | 212.3 | 324.0 | 12 | -623.9 | -572.6 |
| Excluding discipline; disaggregated inst./geo. | 531 | 212.3 | 344.3 | 16 | -656.6 | -588.2 |
| Full model; disaggregated inst./geo. | 531 | 212.3 | 347.5 | 20 | -655.1 | -569.6 |
| **Factor 3: Career development** | | | | | | |
| No job characteristics | 531 | 133.8 | 159.5 | 11 | -297.0 | -249.9 |
| Excluding discipline | 531 | 133.8 | 245.3 | 15 | -460.6 | -396.5 |
| Full model specification | 531 | 133.8 | 264.7 | 19 | -491.4 | -410.1 |
| No job chars; disaggregated inst./geo. | 531 | 133.8 | 159.8 | 12 | -295.5 | -244.2 |
| Excluding discipline; disaggregated inst./geo. | 531 | 133.8 | 246.9 | 16 | -461.8 | -393.4 |
| Full model; disaggregated inst./geo. | 531 | 133.8 | 265.5 | 20 | -491.0 | -405.5 |
| **Factor 4: Outcomes and impact** | | | | | | |
| No job characteristics | 531 | 61.1 | 104.2 | 11 | -186.4 | -139.4 |
| Excluding discipline | 531 | 61.1 | 125.7 | 15 | -221.3 | -157.2 |
| Full model specification | 531 | 61.1 | 142.5 | 19 | -247.0 | -165.8 |
| No job chars; disaggregated inst./geo. | 531 | 61.1 | 106.8 | 12 | -189.6 | -138.3 |
| Excluding discipline; disaggregated inst./geo. | 531 | 61.1 | 128.6 | 16 | -225.1 | -156.7 |
| Full model; disaggregated inst./geo. | 531 | 61.1 | 147.3 | 20 | -254.6 | -169.1 |

## 2.5. Analysis for countries with smaller research systems

The estimated number of active researchers varies significantly across countries and territories, resulting in a highly skewed distribution of country weights in our sample, as shown in Table S8**.** Consequently, some of the results shown here and/or in the main text could be driven by evidence that only applies to a handful of very large countries. On one hand, this would still be representative of the 'global' scenario, in the sense that the rules and criteria set by few countries could have a very large effect globally. But on the other hand, the national research systems can be expected to account for the size of a country, and therefore the rules and criteria that apply in many countries with lower numbers of researchers could be very different and could remain hidden behind the trends set by the large countries.

To investigate this possibility, we conducted a robustness check by re-running all our primary analyses on a subsample that excluded the top 10 countries with the highest estimated number of active researchers. Namely: China, the United States, Japan, South Korea, Germany, Russia, India, France, the United Kingdom, and Taiwan (in orange in Table S8). These countries, listed in decreasing order, account for 72% of the total country weights. Therefore, our robustness check includes 28% of the population of researchers in the 121 countries that are part of our study.

**Table S9. Country weights.** Total weight for each country (equivalent to the sum of the weights of all the institutions in the country) in percentage points. For details about the weighting procedure, see section 1.6. Average country weight = 0.8 p.p.; standard deviation = 2.8 p.p. Note: we do not hold any position on naming conventions. Shorter naming versions were used with the mere objective of simplifying data coding and computational pipelines.

| Country | Weight |
|---|---|
| China | 24.14 |
| United States of America | 15.19 |
| Japan | 6.98 |
| South Korea | 4.72 |
| Germany | 4.63 |
| Russia | 3.95 |
| India | 3.73 |
| France | 3.36 |
| United Kingdom | 3.05 |
| Taiwan | 2.14 |
| Canada | 1.98 |
| Brazil | 1.93 |
| Türkiye | 1.72 |
| Italy | 1.58 |
| Spain | 1.55 |
| Thailand | 1.46 |

| Country | Weight |
|---|---|
| Philippines | 0.20 |
| Tunisia | 0.20 |
| Uzbekistan | 0.19 |
| Bulgaria | 0.16 |
| Kenya | 0.13 |
| Kazakhstan | 0.12 |
| Ethiopia | 0.11 |
| Slovenia | 0.11 |
| Chile | 0.10 |
| Croatia | 0.094 |
| Nigeria | 0.087 |
| Venezuela | 0.084 |
| Iraq | 0.074 |
| Ecuador | 0.073 |
| Jordan | 0.066 |
| Senegal | 0.064 |

| Country | Weight |
|---|---|
| Uganda | 0.014 |
| Tanzania | 0.013 |
| Malta | 0.012 |
| Burkina Faso | 0.011 |
| Chad | 0.011 |
| Dem. Rep. Congo | 0.011 |
| Malawi | 0.011 |
| Lebanon | 0.010 |
| Madagascar | 0.010 |
| Paraguay | 0.010 |
| Sudan | 0.009 |
| Zambia | 0.009 |
| Cambodia | 0.008 |
| Rwanda | 0.008 |
| Benin | 0.008 |
| Niger | 0.007 |

| | |
|---|---|
| Poland | 1.46 |
| Australia | 1.22 |
| Indonesia | 1.11 |
| Netherlands | 1.07 |
| Pakistan | 1.00 |
| Egypt | 0.93 |
| Sweden | 0.87 |
| Vietnam | 0.77 |
| Israel | 0.77 |
| Argentina | 0.59 |
| Mexico | 0.46 |
| Algeria | 0.46 |
| Denmark | 0.46 |
| Hungary | 0.45 |
| Greece | 0.45 |
| Finland | 0.44 |
| Morocco | 0.41 |
| Hong Kong | 0.34 |
| South Africa | 0.29 |
| Belarus | 0.27 |
| Ireland | 0.27 |
| Saudi Arabia | 0.26 |
| United Arab Emirates | 0.25 |
| Malaysia | 0.25 |
| Ukraine | 0.22 |

| | |
|---|---|
| Bangladesh | 0.062 |
| Estonia | 0.054 |
| Colombia | 0.047 |
| Kyrgyzstan | 0.041 |
| Cameroon | 0.033 |
| Palestine | 0.032 |
| Peru | 0.031 |
| Ghana | 0.030 |
| Uruguay | 0.028 |
| Cuba | 0.027 |
| Moldova | 0.027 |
| Qatar | 0.027 |
| Iceland | 0.026 |
| Sri Lanka | 0.023 |
| Cyprus | 0.022 |
| Costa Rica | 0.021 |
| Bolivia | 0.020 |
| Côte d'Ivoire | 0.020 |
| Syrian Arab Rep. | 0.020 |
| Nepal | 0.018 |
| Armenia | 0.018 |
| Guinea | 0.017 |
| Zimbabwe | 0.017 |
| Tajikistan | 0.015 |
| Bosnia & Herzegovina | 0.014 |

| | |
|---|---|
| Mauritius | 0.007 |
| Kuwait | 0.007 |
| Angola | 0.007 |
| Mali | 0.007 |
| Yemen | 0.007 |
| Congo | 0.006 |
| Burundi | 0.006 |
| Bahrain | 0.006 |
| El Salvador | 0.005 |
| Botswana | 0.005 |
| Albania | 0.004 |
| Gabon | 0.004 |
| Togo | 0.004 |
| Namibia | 0.004 |
| Guatemala | 0.003 |
| Guinea-Bissau | 0.003 |
| Panama | 0.002 |
| Brunei Darussalam | 0.0013 |
| Central African Rep | 0.0009 |
| Mauritania | 0.0007 |
| Djibouti | 0.0004 |
| Equatorial Guinea | 0.0003 |
| Comoros | 0.0001 |

On this dataset, following the same procedures done for the whole sample (Methods sect 3.2), we conducted a factor analysis on the matrix of polychoric correlations among all policy criteria, using the same structure of weights. This yielded four factors with eigenvalues greater than 2, which cumulatively explained 57% of the observed variance. These factors were rotated using the oblimin oblique method, and the resulting factor loadings are presented in Table S9.

By and large, we obtain factors that very closely reflect those from the full sample. This sustains two key findings: First, a main substantive result – that policy documents tend to focus on coherent criteria within a country, regardless of e.g. discipline or track – is a truly global phenomenon and not a characteristic of a few large countries. Second, policy documents generally show similar tendencies in the co-occurrence of criteria, making it possible to retain the same interpretation of the factors for this sub-dataset (Most of the differences observed for factor loadings in Table S9 and Figure 4 involve loadings close to 0.3 in absolute value, our threshold for visualisation in the table, which are not displayed due to their minimal impact)

**Table S10. Factor loadings in countries with less influential research systems.** Performed with the same specifications as for Figure 4, but without the effect of the 10 most influential countries in our sample. Blanks cells denote loadings smaller than 0.3 in absolute value.

| CRITERIA | Output metrics | Visibility & Engagement | Professional development | Outcomes & Impact | *Uniqueness* |
|---|---|---|---|---|---|
| N. of publications | 0.682 | | | | *0.4123* |
| Recent publications | 0.378 | 0.3895 | | -0.5299 | *0.3792* |
| Patents | 0.657 | | | 0.4493 | *0.4212* |
| Citations | 0.4566 | | 0.4256 | -0.4755 | *0.3798* |
| Journal indexing | 0.8841 | | | | *0.1821* |
| Number of authors | 0.8619 | | | | *0.2568* |
| Authorship order | 0.7279 | | | | *0.2602* |
| Non-metric journal quality | -0.3468 | 0.7604 | | -0.3749 | *0.2467* |
| Role of authors | | 0.3922 | 0.5019 | -0.3868 | *0.4072* |
| Nonmetric publication quality | -0.4369 | 0.3218 | 0.3917 | | *0.3822* |
| Social Impact | | 0.5907 | 0.3856 | | *0.3616* |
| Farsight | -0.4111 | 0.3827 | 0.4096 | | *0.3866* |
| Funding | | | 0.4525 | | *0.7686* |
| Collaborations | -0.3356 | | 0.6163 | | *0.2679* |
| Experience abroad | | -0.4792 | 0.604 | | *0.5202* |
| Presentations | 0.5423 | | 0.3017 | | *0.6059* |
| Professional development | | | -0.3141 | 0.6616 | *0.4992* |
| Memberships | 0.4024 | 0.3363 | | 0.5264 | *0.4151* |
| Professional Titles | | | | | *0.8625* |
| Invited positions | | 0.4003 | 0.521 | | *0.4637* |
| Awards | 0.5332 | | 0.554 | | *0.4277* |
| Commercialisation/Consultancy | | | 0.38 | | *0.7948* |
| Service to the Profession | | | 0.4043 | 0.6572 | *0.2814* |
| Mentoring | | | | 0.6749 | *0.423* |
| Teaching | | 0.6969 | | | *0.4175* |
| Administrative roles | | 0.7128 | | 0.4734 | *0.2194* |
| Community service | | 0.7296 | | | *0.3479* |
| Serving period | 0.3848 | 0.4178 | -0.4774 | | *0.5234* |
| Interdisciplinarity | | | 0.5314 | | *0.5894* |
| Ethics and integrity | | 0.7222 | | | *0.3858* |

Similarly to the main analysis, we conducted separate regressions on the factors, using the same explanatory variables discussed in section 2.4 and in Figure 5. As shown in Table S10, several results in this sample replicate those found for the whole sample. Here, we confirmed that characteristics of the position do not seem to frequently exert a statistically significant impact on the four factors (and therefore on the latent characteristics of the policy documents). First, even fewer differences emerge between documents that only apply for promotions to full professor and those that apply to lower ranks too: in this smaller sample, only visibility is slightly more often requested in documents that apply to full professors only. Second, few differences emerge among tracks, with clinical tracks more often considering outcomes and societal impact. Third, we confirm that the differences in the criteria across disciplines are very few: similar to the full sample, they concern engineering, and for this subsample it also includes the social sciences, which less often consider outcomes and impact when compared to general policies.

Naturally, we also found slightly different results in the sub-sample concerning country and institution characteristics. Perhaps, the main difference between the results in the full sample and the sub-sample is that in the latter we do not find statistically significant differences between the Global North and South, once controlling for all the other covariates included in the regressions. More specifically, when more finely distinguishing, by both, region and type of document, we confirm that national policies focus on metrics significantly more than institutional policies, which is consistently true for countries in the Global South in this analysis. On the other hand, we no longer find that professional development is a special concern for institutions in the Global South, possibly because this association is more related to smaller research systems. As in the main analysis, we find some statistically significant differences between continents. Additionally, we now find fewer statistically significant differences across country income levels - in particular, upper-middle income countries do not emerge as significantly relying on any policy criteria more or less often than high-income countries.

Overall, most differences emerge in the second and third factors (visibility & engagement, and professional development), where the factor analysis also resulted in slightly different definitions in terms of factor loadings of the individual policy criteria. As mentioned earlier, occasional discrepancies within an overall similar set of results do not imply that either the analysis on the full sample or the present one is biassed or incorrect. Instead, they highlight the crucial influence of a few very large countries in shaping certain details of our findings. An in-depth investigation of these aspects goes beyond the scope of the present study, as it would require a sample of institutions representative at the country level.

**Table S11. Regression analysis in countries with less influential research systems**. Regression analysis performed as per Figure 5 for a subsample without the effect of the 10 countries with larger weights in our sample. Here are the regression results on the four factors in the reduced sample of countries obtained by excluding the ten countries with the

largest country weights. All specifications and definitions are as in Table S7. Heteroskedasticity-robust standard errors in brackets and in grey font, clustered by institution or agency. *** denotes p < 0.01, **: p < 0.05, and *: p < 0.1.

| CLASSES | Factor 1 Output metrics | | Factor 2 Visibility, engagement | | Factor 3 Career development | | Factor 4 Outcomes & impact | |
|---|---|---|---|---|---|---|---|---|
| | [1] | [2] | [1] | [2] | [1] | [2] | [1] | [2] |
| ***Global Region and Policy Scope (Independent)*** | | | | | | | | |
| National policies | 0.156*** | - | 0.0365 | - | -0.0227 | - | -0.0671 | - |
| | [0.0525] | - | [0.0460] | - | [0.0339] | - | [0.0414] | - |
| Global South | 0.151 | - | -0.0827 | - | -0.0688 | - | -0.0301 | - |
| | [0.128] | - | [0.101] | - | [0.0910] | - | [0.0773] | - |
| ***Global Region and Policy Scope (clustered)*** | | | | | | | | |
| National policies, Global North | - | 0.217*** | - | -0.0637 | - | -0.0566 | - | -0.0513 |
| | - | [0.0707] | - | [0.0617] | - | [0.0503] | - | [0.0450] |
| Institutional policies, Global South | - | 0.165 | - | -0.106 | - | -0.0766 | - | -0.0265 |
| | - | [0.126] | - | [0.101] | - | [0.0915] | - | [0.0790] |
| National policies, Global South | - | 0.260* | - | 0.0320 | - | -0.0651 | - | -0.110 |
| | - | [0.155] | - | [0.104] | - | [0.105] | - | [0.0817] |
| ***Disciplines*** | | | | | | | | |
| Natural sciences | 0.0836 | 0.0798 | -0.0535 | -0.0472 | -0.0365 | -0.0343 | -0.0215 | -0.0225 |
| | [0.0644] | [0.0617] | [0.0611] | [0.0589] | [0.0420] | [0.0419] | [0.0431] | [0.0425] |
| Medicine & health sciences | 0.0434 | 0.0418 | -0.00852 | -0.00591 | -0.0615 | -0.0606 | -0.0876** | -0.0880** |
| | [0.113] | [0.117] | [0.0502] | [0.0509] | [0.0664] | [0.0657] | [0.0415] | [0.0406] |
| Social sciences & humanities | 0.00154 | 0.00400 | -0.0601 | -0.0642 | 0.0159 | 0.0145 | -0.141*** | -0.140*** |
| | [0.0447] | [0.0430] | [0.0454] | [0.0412] | [0.0427] | [0.0453] | [0.0416] | [0.0417] |
| Engineering & technology | 0.0591 | 0.0488 | -0.218*** | -0.201*** | 0.0147 | 0.0204 | -0.0978 | -0.100 |
| | [0.0555] | [0.0537] | [0.0435] | [0.0513] | [0.0642] | [0.0660] | [0.0614] | [0.0614] |
| ***Tracks*** | | | | | | | | |
| Research track | -0.00572 | 0.0139 | 0.00338 | -0.0289 | 0.0361 | 0.0252 | 0.0500 | 0.0550 |
| | [0.0567] | [0.0595] | [0.0501] | [0.0455] | [0.0397] | [0.0400] | [0.0735] | [0.0772] |
| Teaching track | -0.0730 | -0.0694 | 0.0738* | 0.0678 | 0.0158 | 0.0137 | -0.0252 | -0.0242 |
| | [0.0780] | [0.0811] | [0.0406] | [0.0417] | [0.0338] | [0.0342] | [0.0338] | [0.0347] |
| Clinical or other | -0.0971 | -0.0952 | -0.00348 | -0.00663 | 0.0737 | 0.0726 | 0.0902** | 0.0907** |
| | [0.0973] | [0.0975] | [0.0783] | [0.0771] | [0.0781] | [0.0778] | [0.0357] | [0.0354] |
| ***Continents*** | | | | | | | | |
| Africa | 0.0748 | 0.0913 | 0.254** | 0.227** | 0.0384 | 0.0292 | 0.0172 | 0.0215 |
| | [0.142] | [0.143] | [0.107] | [0.102] | [0.0887] | [0.0919] | [0.0932] | [0.0900] |
| Asia | -0.0180 | 0.0174 | 0.162* | 0.104 | 0.0205 | 0.000880 | -0.0763 | -0.0672 |
| | [0.105] | [0.108] | [0.0977] | [0.0931] | [0.0572] | [0.0621] | [0.0862] | [0.0785] |
| Latin America | -0.161 | -0.162 | 0.143 | 0.144 | -0.00115 | -0.000802 | 0.0666 | 0.0664 |
| | [0.105] | [0.101] | [0.117] | [0.115] | [0.0709] | [0.0734] | [0.0972] | [0.0970] |
| North America | 0.0774 | 0.0996 | 0.154** | 0.117 | 0.0994 | 0.0871 | 0.00520 | 0.0110 |
| | [0.0725] | [0.0753] | [0.0767] | [0.0782] | [0.0974] | [0.0983] | [0.0339] | [0.0330] |
| Oceania | 0.0854 | 0.102 | 0.0801 | 0.0529 | 0.0904* | 0.0812 | -0.0241 | -0.0198 |
| | [0.0783] | [0.0804] | [0.0665] | [0.0667] | [0.0514] | [0.0526] | [0.0536] | [0.0523] |
| ***Economic Status (Income level)*** | | | | | | | | |
| Upper-middle income | 0.124 | 0.140 | 0.0707 | 0.0446 | -0.0409 | -0.0497 | 0.0616 | 0.0657 |
| | [0.0877] | [0.0868] | [0.0588] | [0.0559] | [0.0763] | [0.0739] | [0.0537] | [0.0502] |
| Lower-middle income | -0.0168 | 0.00853 | -0.0946 | -0.136** | -0.134 | -0.148* | 0.0523 | 0.0589 |
| | [0.106] | [0.101] | [0.0625] | [0.0648] | [0.0856] | [0.0803] | [0.0532] | [0.0591] |
| Low-income | -0.0903 | -0.0720 | 0.00665 | -0.0234 | -0.257*** | -0.267*** | -0.0708 | -0.0661 |
| | [0.131] | [0.136] | [0.0751] | [0.0729] | [0.0930] | [0.0878] | [0.0689] | [0.0730] |

| | | | | | | | | |
|---|---|---|---|---|---|---|---|---|
| **Criteria specific for full professor** | | | | | | | | |
| Policy specific for full professor | 0.0165 | 0.0180 | 0.0866* | 0.0842* | 0.0295 | 0.0287 | -0.00419 | -0.00381 |
| | [0.0562] | [0.0514] | [0.0458] | [0.0446] | [0.0422] | [0.0417] | [0.0310] | [0.0312] |
| ***Regression Parameters*** | | | | | | | | |
| Constant | 0.405*** | 0.381*** | 0.454*** | 0.493*** | 0.569*** | 0.582*** | 0.626*** | 0.619*** |
| | [0.0559] | [0.0535] | [0.0491] | [0.0554] | [0.0383] | [0.0391] | [0.0368] | [0.0355] |
| Observations | 416 | 416 | 416 | 416 | 416 | 416 | 416 | 416 |
| R-squared | 0.402 | 0.413 | 0.281 | 0.327 | 0.300 | 0.305 | 0.337 | 0.339 |

## 3. REFERENCES


**[1]** J. P Alperin, C. Muñoz Nieves, L. A. Schimanski, G. E. Fischman, M. T. Niles, & E. C. McKiernan (2019), "*Meta-Research: How significant are the public dimensions of faculty work in review, promotion and tenure documents?*". eLife 8, e42254. DOI: 10.7554/eLife.42254

**[2]** Lim, B. H., D'Ippoliti, C., Dominik, M., Vermeir, K., Chong, K.-K., Backes, M., Palomo, I., Molnar, A., Hernández-Mondragón, A., dela Cruz, T. E., Mondello, S., López-Vergès, S., Coussens, A., Enany, S., Kumar, P., Jambrak, A. R., Siciliano, V., Bhadra, A., Bassioni, G., Xuereb, A., & Flores Bueso, Y. (2023). *A global assessment of academic promotion criteria: What really counts?* Research Square. https://doi.org/10.21203/rs.3.rs-3011208/v1

**[3]** United Nations. (n.d.). United Nations Geoscheme. Retrieved from https://unstats.un.org/unsd/methodology/m49/

**[4]** World Bank, Country Classification: World Bank Country and Lending Groups. https://datahelpdesk.worldbank.org/knowledgebase/articles/906519-world-bank-country-and-lending-groups, Fiscal Year 2012. (Accessed: 22 Jan 2022)

**[5]** O. Hoffmeister (2020), "Development Status as a Measure of Development", United Nations Conference on Trade and Development (UNCTAD) Research Papers, 46. DOI: 10.18356/a29d2be8-en

**[6]** Working Party of National Experts on Science and Technology Indicators, (2007). Revised field of science and technology (FoS) classification in the research manual. Organisation for Economic Co-operation and Development (OECD), DSTI/EAS/STP/NESTI(2006)19/FINAL.

**[7]** United Nations Educational, Scientific, and Cultural Organization (UNESCO). (2023). UIS Data Centre. Retrieved from UIS website: http://data.uis.unesco.org/index.aspx?queryid=3685

**[8]** UNESCO Institute for Statistics (UIS), "Researchers in R&D (per million people)", [SP.POP.SCIE.RD.P6]. UIS Bulk Data Download Service. https://apiportal.uis.unesco.org/bdds (Accessed: 27 Nov 2023)

**[9]** United Nations Educational, Scientific and Cultural Organization (UNESCO) (2016), "UNESCO Science Report: Towards 2030". Second revised edition. UNESCO Publishing, Paris. DOI: 10.18356/9789210059053

**[10]** United Nations Educational, Scientific and Cultural Organization (UNESCO) (2021), "UNESCO Science Report: the Race Against Time for Smarter Development. S. Schneegans, T. Straza, & J. Lewis (eds). UNESCO Publishing, Paris. DOI: 10.18356/9789210058575

**[11]** SDG Cell, Bangladesh Bureau of Statistics, "Indicators 9.5.2: Researchers (in full-time equivalent) per million inhabitants (2022)", in: "SDG Tracker – Bangladesh's Development Mirror", Available at: https://sdg.gov.bd/page/indicator-wise/1/101/3/0 (Accessed: 29 Feb 2024).

**[12]** National Statistical Committee of the Kyrgyz Republic, "Indicator 9.5.2 - Researchers (in full-time equivalent) per million inhabitants (2022)", in: "Sustainable Development Goals in the Kyrgyz Republic". Available at:

https://sustainabledevelopment-kyrgyzstan.github.io/en/9-5-2/#:~:text=Year%2C%202017%2C%202018%2C%202019%2C%202020%2C%20Value%2C%20524%2C%20555%2C%20527%2C%20534%2C (Accessed: 29 Feb 2024)

**[13]** Statista, "Number of research personnel per 10,000 population in Taiwan from 2011 to 2021". https://www.statista.com/statistics/324708/taiwan-number-of-researchers-per-10000-population/ (Accessed: 29 Feb 2024)

**[14]** J. P. Stevens (2002). Applied multivariate statistics for the social sciences (5th ed.). Routledge.
