# Supplementary material for: Regional and institutional trends in assessment for academic promotion
Source: Nature. 2025 Jan 22;638(8050):459–68. doi: 10.1038/s41586-024-08422-9 (PMC11821531; doi:10.1038/s41586-024-08422-9)
Supplement: Supplementary file 2 — Reporting Summary [file 41586_2024_8422_MOESM2_ESM.pdf]

nature portfolio

Corresponding author(s): YENSI FLORES BUESO

Last updated by author(s): Oct 23, 2024

Reporting Summary

Nature Portfolio wishes to improve the reproducibility of the work that we publish. This form provides structure for consistency and transparency in reporting. For further information on Nature Portfolio policies, see our Editorial Policies and the Editorial Policy Checklist.

Statistics

For all statistical analyses, confirm that the following items are present in the figure legend, table legend, main text, or Methods section.

n/a	Confirmed	
☐	☒	The exact sample size (*n*) for each experimental group/condition, given as a discrete number and unit of measurement
☐	☒	A statement on whether measurements were taken from distinct samples or whether the same sample was measured repeatedly
☐	☒	The statistical test(s) used AND whether they are one- or two-sided<br>*Only common tests should be described solely by name; describe more complex techniques in the Methods section.*
☐	☒	A description of all covariates tested
☐	☒	A description of any assumptions or corrections, such as tests of normality and adjustment for multiple comparisons
☐	☒	A full description of the statistical parameters including central tendency (e.g. means) or other basic estimates (e.g. regression coefficient) AND variation (e.g. standard deviation) or associated estimates of uncertainty (e.g. confidence intervals)
☐	☒	For null hypothesis testing, the test statistic (e.g. *F*, *t*, *r*) with confidence intervals, effect sizes, degrees of freedom and *P* value noted<br>*Give P values as exact values whenever suitable.*
☒	☐	For Bayesian analysis, information on the choice of priors and Markov chain Monte Carlo settings
☒	☐	For hierarchical and complex designs, identification of the appropriate level for tests and full reporting of outcomes
☒	☐	Estimates of effect sizes (e.g. Cohen's *d*, Pearson's *r*), indicating how they were calculated

Our web collection on statistics for biologists contains articles on many of the points above.

Software and code

Policy information about availability of computer code

Data collection	Data was collected using Google Sheets and compiled into a single file downloaded and transformed in Microsoft Excel 16 (Mac) to a CSV file that was the input for the software for analysis. The raw data was shared in a csv format in CodeOcean. Both, raw and clean datasets are shared here: https://doi.org/10.6084/m9.figshare.23272175.
Data analysis	Data was analysed using Stata17 and plotted using python3 pandas and matplotlib. The panels were assembled and formated using Adobe Ilustrator 2024. The code for data analysis was shared in CodeOcean. https://codeocean.com/capsule/0942594/tree .

For manuscripts utilizing custom algorithms or software that are central to the research but not yet described in published literature, software must be made available to editors and reviewers. We strongly encourage code deposition in a community repository (e.g. GitHub). See the Nature Portfolio guidelines for submitting code & software for further information.

Data

Policy information about availability of data

All manuscripts must include a data availability statement. This statement should provide the following information, where applicable:

- Accession codes, unique identifiers, or web links for publicly available datasets
- A description of any restrictions on data availability
- For clinical datasets or third party data, please ensure that the statement adheres to our policy

We have included a Data availability statement and we share all the data produced by this study.

Research involving human participants, their data, or biological material

Policy information about studies with human participants or human data. See also policy information about sex, gender (identity/presentation), and sexual orientation and race, ethnicity and racism.

Reporting on sex and gender	N/A
Reporting on race, ethnicity, or other socially relevant groupings	N/A
Population characteristics	N/A
Recruitment	N/A
Ethics oversight	We are not dealing with any humnan participants, their data or biological material. Our study is limited to the analysis of policies that are developed by instituions and may impact humans but do not directly relate to them.

Note that full information on the approval of the study protocol must also be provided in the manuscript.

Field-specific reporting

Please select the one below that is the best fit for your research. If you are not sure, read the appropriate sections before making your selection.

☐ Life sciences ☒ Behavioural & social sciences ☐ Ecological, evolutionary & environmental sciences

For a reference copy of the document with all sections, see nature.com/documents/nr-reporting-summary-flat.pdf

Behavioural & social sciences study design

All studies must disclose on these points even when the disclosure is negative.

Study description	We conducted a cross-sectional quantitative analysis by examining the assessment criteria used in promotion policies. We systematically identified and analysed promotion criteria, comparing differences and similarities across disciplines, fields, tracks, types of institutions, and countries, considering their socioeconomic contexts. We solely recorded the presence or absence of subjectively chosen distinct assessment criteria in available documents describing promotion policies, without any regard to the process of hiring or promotion.
Research sample	The study analyzed 532 promotion policies from 190 academic institutions and 58 government agencies across 121 countries. The sample was chosen to maximize global diversity and capture a wide range of institutional practices, focusing on full professor promotion policies as is the most senior position that can be comparable between institutions in different countries. While not representative for all academic and national organisations, our sample has the power to sustain our claims (see SI sect 2.3)
Sampling strategy	The documents were obtained by global snowball sampling originating from the GYA network rather than as a randomised sample across all relevant institutions or authorities in the world, and our sample is not representative at country level. Our sample size was informed by power analysis, which as seen in SI section 2.3 confirm that our sample has the power to sustain our claims.
Data collection	Policies were obtained from public websites, academic networks, and official requests. The collected documents were analyzed to identify the presence or absence of specific criteria related to the promotion of full professors. Data coding was performed by members of the team using a standardised template, definitions, protocols and supporting materials.
Timing	Data study was initiated in 2016 with a pilot study to define the methodology. Documents were collected since then. However, 83% documents in the version published of this manuscript were obtained in 2022/2023 to ensure that they are still valid.
Data exclusions	We excluded data from documents that did not provide clear or comprehensive information about promotion criteria. Additionally, we limited our analysis to promotion policies for full professors, excluding documents that did not cover this role or focused on career tracks unrelated to both research and teaching, to ensure consistency and comparability of different data points. We excluded a total of 24 policies from our study. Throughout the process we documented the exclusion of 59 policies.
Non-participation	n/a
Randomization	n/a

Reporting for specific materials, systems and methods

We require information from authors about some types of materials, experimental systems and methods used in many studies. Here, indicate whether each material, system or method listed is relevant to your study. If you are not sure if a list item applies to your research, read the appropriate section before selecting a response.

Materials & experimental systems

n/a	Involved in the study
☒	☐ Antibodies
☒	☐ Eukaryotic cell lines
☒	☐ Palaeontology and archaeology
☒	☐ Animals and other organisms
☒	☐ Clinical data
☒	☐ Dual use research of concern
☒	☐ Plants

Methods

n/a	Involved in the study
☒	☐ ChIP-seq
☒	☐ Flow cytometry
☒	☐ MRI-based neuroimaging

Plants

Seed stocks	n/a
Novel plant genotypes	n/a
Authentication	n/a
